# Mansonone G and its derivatives exhibit membrane permeabilizing activities against bacteria

**DOI:** 10.1371/journal.pone.0273614

**Published:** 2022-09-01

**Authors:** Htut Htut Htoo, Nhung Ngo Thi Tuyet, Kittiporn Nakprasit, Chanat Aonbangkhen, Vorrapon Chaikeeratisak, Warinthorn Chavasiri, Poochit Nonejuie

**Affiliations:** 1 Institute of Molecular Biosciences, Mahidol University, Nakhon Pathom, Thailand; 2 Center of Excellence in Natural Products Chemistry (CENP), Department of Chemistry, Chulalongkorn University, Bangkok, Thailand; 3 Department of Biochemistry, Chulalongkorn University, Bangkok, Thailand; Universidade Estadual de Ponta Grossa, BRAZIL

## Abstract

In an era where the rate of bacteria evolving to be resistant to clinically-used antibiotics far exceeds that of antibiotic discovery, the search for new sources of antibacterial agents has expanded tremendously. In recent years, interest in plant-based natural products as promising sources of antibacterial agents has taken an upward trend. Mansonones, botanically-derived naphthoqionones, having many uses in Asian traditional medicine–including anti-infective roles–have sparked interest as a possible source of antibacterial agents. Here, we show that mansonone G, extracted from *Mansonia gagei* Drumm. heartwoods, possessed antibacterial activities towards *Bacillus subtilis*, *Staphylococcus aureus* and *Escherichia coli* lptD4213, inhibiting the growth of the bacteria at 15.6 μM, 62.5 μM and 125 μM, respectively. Fourteen derivatives of mansonone G were synthesized successfully and were found to have a similar antibacterial spectrum to that of the parent compound, with some derivatives possessing improved antibacterial activities. Bacterial cytological profiling analysis showed that mansonone G harbors membrane permeabilizing activities against *B*. *subtilis and E*. *coli* lptD4213. Temporal analysis of SYTOX Green staining among individual cells showed that mansonone G rapidly permeabilized bacterial membrane within 10 min, with SYTOX Green intensity reaching 13-fold above that of the control. Collectively, these findings highlight the importance of mansonone G and its derivatives as potential antibacterial agents, paving the way for further modifications in order to improve their antibacterial spectrum.

## Introduction

The introduction of salvarsan, the first antibiotic, in 1910 revolutionized medicine, not only in saving countless number of lives but also in making procedures like cancer treatment and organ transplant a possibility [1]. The golden era of antibiotic discovery began in 1928, with penicillin, and finding its peak in the 1950s, adding new classes of antibiotics to the fast-expanding repertoire of antibacterial agents every few years [1, 2]. Since then, with the decline of novel classes of antibiotics being discovered, coupled with the emergence of multi-drug resistant (MDR) bacteria, focus was shifted to create broad-spectrum antibiotics [3]. Unfortunately, liberal use of antibiotics has resulted in resistance at alarming rates, to the extent that resistance is developing within a few years after the production and release of a new antibiotic [4].

Antimicrobials can be obtained from a number of natural sources such as from land and marine plants, seaweed, fungi and from bacteria themselves [5]. The best example of antimicrobial-producing bacteria would be *Streptomyces*, a Gram-positive, terrestrial, and aquatic bacteria, responsible for producing almost two-thirds of the antibiotics used to date [6]. Compounds and metabolites from *Streptomyces* have been extensively explored and, as is the case with most natural resources, have now been exhausted, and thus the hunt for novel antimicrobial agents continues. Compounds under research and development for use as antimicrobial agents in the clinical setting are, in many instances, improved variants of marketed or existing compounds and drugs that show promising activities [7]. With the exhaustion of antimicrobial sources and the threat from MDR bacteria casting a dark cloud over the future of medicine, plant-derived antimicrobial compounds, and their myriad of secondary metabolites–used for centuries around the world in traditional medicine–have gained increasing consideration and are being scrutinized as alternatives to antibiotics [8].

*Mansonia gagei* Drumm, native to Thailand [9], goes by several different local names—chan-cha-mod, chan-hom, chan-khao, or chan-pa-ma [10]. Its most useful part has been identified as its heartwoods and has found a use in traditional medicine as an antidepressant, antiemetic, cardiac stimulant and as a refreshment agent [11]. Compounds from this botanical source, including the 1,2-naphthoquinones—mansonones—were also found to possess antifungal, antioxidant and larvicidal properties [12]. Mansonone G, a naturally occurring *o*-naphthoquinone (5% of CH_2_Cl_2_ extract), has been studied by many and found to show antifungal [13] and anticancer [9, 14, 15] properties. Furthermore, cytotoxicity tests of mansonone G in HCT-116, HepG2, MCF-7 and HeLa cell lines also provided promising results [16] highlighting its potency as a good candidate for further drug development.

Apart from the myriad of bioactivities of mansonone G, a previous study had shed some light onto antibacterial activity of the compound and its allyl and prenyl ether derivatives highlighting the importance of medicinal chemistry that could potentially broaden and improve antibacterial activity of the compound against various bacteria in this dire situation of antibiotic resistance [17]. Spurred by the promising antibacterial activity of mansonone G and its derivatives, here in this study, we further examined mansonone G and other derivatives for their antibacterial spectrum, activities, and mechanism of action in both Gram-positive and Gram-negative bacteria including some of the most critical human pathogens such as *Staphylococcus aureus*, *Acinetobacter baumannii* and *Pseudomonas aeruginosa*. By performing a phenotypic profiling assay, we showed that mansonone G exerts its antibacterial activity against an LPS-defected *E*. *coli* and *Bacillus subtilis* via a membrane permeabilizing mechanism.

## Results

### Mansonone G inhibits the growth of *S*. *aureus*, *B*. *subtilis* and an LPS-defected *E*. *coli*

Natural products have, in recent years, become one of the preferred choices to screen in the search for novel antimicrobial agents [4]. The discovery of mansonone G harboring an array of bioactivities [18] together with the finding in the previous study that certain analogues of mansonone G were bactericidal against some Gram-positive and Gram-negative bacteria [17], compelled us to further investigate the antibacterial spectrum of mansonone G. We screened for the antibacterial activity of the compound against both Gram-positive and Gram-negative bacteria including human pathogens; *S*. *aureus*, *A*. *baumannii*, *P*. *aeruginosa* and *V*. *parahaemolyticus*. Our findings indicated that, while mansonone G exhibited antibacterial activity against Gram-positive *S*. *aureus* and *B*. *subtilis*, the compound did not display any growth inhibitory effect on the Gram-negative pathogens tested as shown in Table 1, suggesting that mansonone G poorly exhibits antibacterial activity against Gram-negative bacteria. However, mansonone G displayed activity against LPS-defected *E*. *coli* strain lptD4213, a mutant strain which was previously shown to be more susceptible to antibiotics including some of the Gram-positive specific drugs such as vancomycin, due to the lack of lipopolysaccharide on the cell surface [19].

**Table 1:** Minimal inhibitory concentration of mansonone G and its derivatives.

Compounds	Minimal Inhibitory Concentration (mM)							
	B. subtilis (PY79)	S. aureus (ATCC29213)	E. coli (lptD4213)	E. coli (MC4100)	E. coli (ATCC25922)	A. baumannii (ATCC19606)	P. aerugenosa (PA01)	V. parahaemolyticus (AHPND)
Mansonone G	15.6	62.5	125	> 250	> 250	> 250	> 250	> 250
5	7.8	15.6	31.25	> 250	> 250	> 250	> 250	> 250
6	> 125	> 125	> 250	> 250	> 250	> 250	> 250	> 250
7	31.25	31.25	> 250	> 250	> 250	> 250	> 250	> 250
8	> 125	> 125	> 250	> 250	> 250	> 250	> 250	> 250
9	7.8	15.6	> 250	> 250	> 250	> 250	> 250	> 250
10	15.6	15.6	62.5	> 250	> 250	> 250	> 250	> 250
11	> 125	> 125	> 250	> 250	> 250	> 250	> 250	> 250
12	> 125	> 125	> 250	> 250	> 250	> 250	> 250	> 250
13	> 125	> 125	> 250	> 250	> 250	> 250	> 250	> 250
14	> 125	> 125	> 250	> 250	> 250	> 250	> 250	> 250
15	31.25	31.25	> 250	> 250	> 250	> 250	> 250	> 250
16	7.8	15.6	> 250	> 250	> 250	> 250	> 250	> 250
17	15.6	15.6	125	> 250	> 250	> 250	> 250	> 250
18	15.6	31.25	> 250	> 250	> 250	> 250	> 250	> 250

### Antibacterial activities of mansonone G derivatives

**Fig 1:**
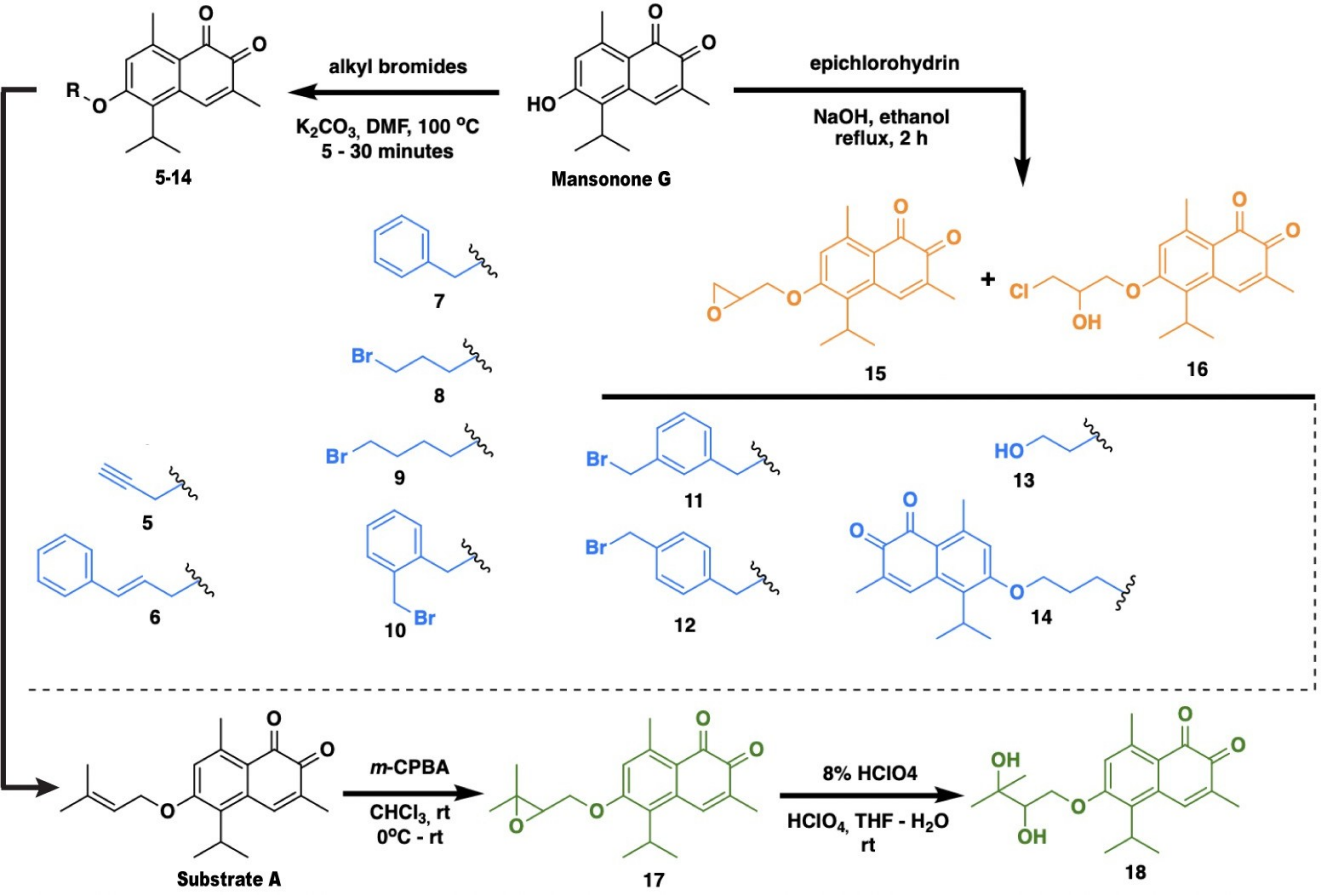
Schematics showing general procedure to synthesize ether derivatives of mansonone G.

The outer membrane of the Gram-negative bacterium functions as a protective barrier, limiting the influx of surrounding molecules; thus, many active molecules fail to exert their antibacterial activity [20]. As part of this study, 14 semisynthetic derivatives of mansonone G (Fig 1 and Table 1) were included in the screening, to test whether the modification could lead to activity improvement against the bacteria. Our findings indicated that in *B*. *subtilis*, 6 out of 14 and in *S*. *aureus*, 8 out of 14 compounds were able to inhibit the growth of these bacteria at concentrations equal to, or lower than, that of the parent mansonone G (Table 1). Three of the derivatives possessed antibacterial activity against *E*. *coli* lptD4213, with compound 5 and 10 showing higher efficiency, by inhibiting bacteria growth at a lower concentration than mansonone G. However, similar to that seen in mansonone G, none of the derivatives showed the ability to inhibit the growth of any of the Gram-negative bacteria screened. These findings have indicated that some of the modifications done to mansonone G have resulted in derivatives possessing higher bactericidal efficiencies than the parent compound in Gram-positive bacteria and an LPS-defected *E*. *coli* strain but not in other Gram-negative bacteria.

### Bacterial cytological profiling (BCP) reveals that mansonone G and its derivatives possess membrane permeabilizing activities

**Fig 2:**
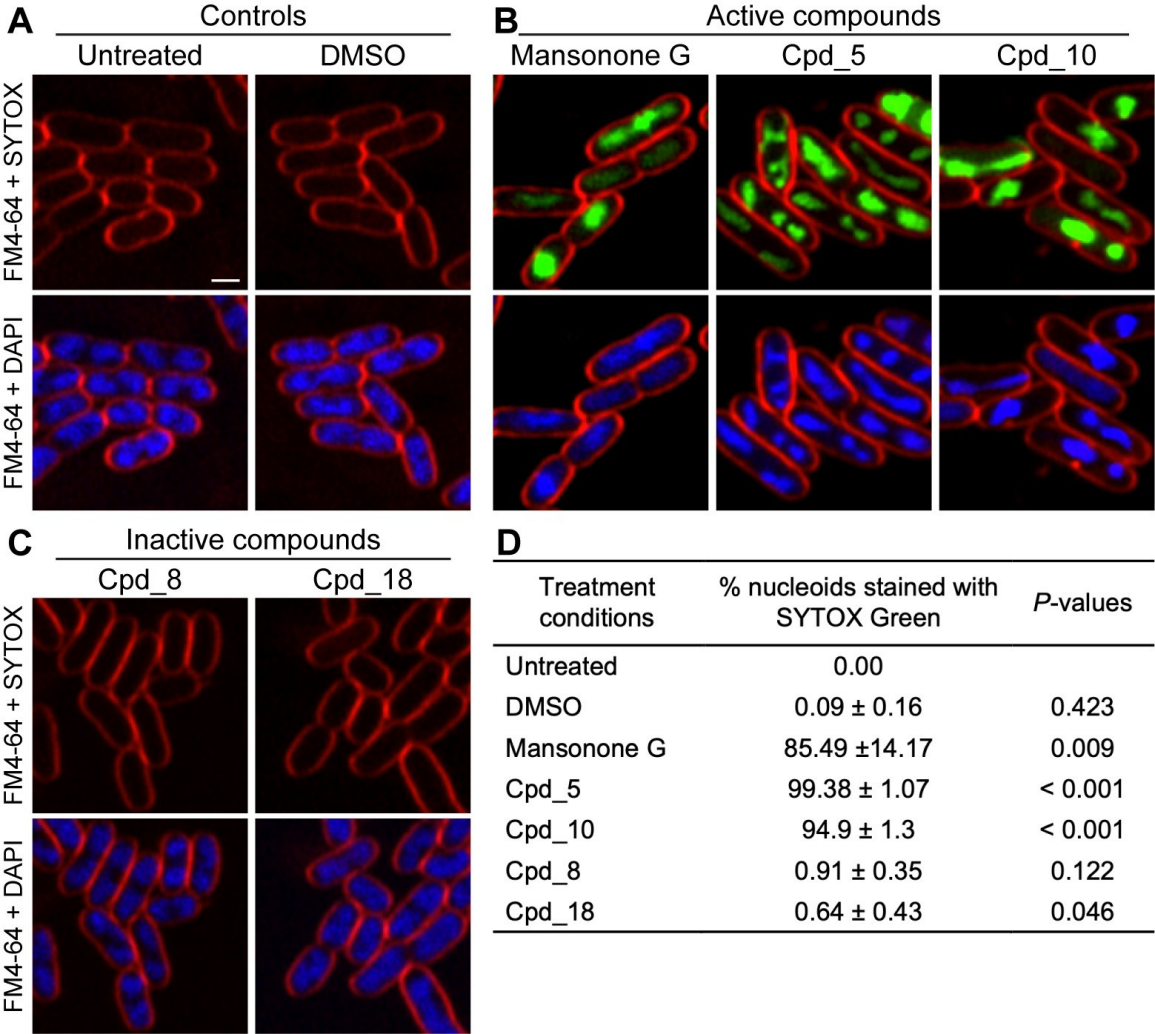
Mansonone G and its derivatives showing membrane permeabilizing activity in *E*. *coli* lptD4213. *E*. *coli* lptD4213 cells were treated for 120 minutes with compounds and then stained with 2 μg/ml FM4-64 (red), 4 μg/ml DAPI (blue) and 0.5 μM SYTOX Green (Green). Upper panels show FM4-64 and SYTOX Green while lower panels show FM4-64 and DAPI, for treatment conditions; (A) untreated control and 0.25% v/v DMSO-treated control, (B) active compounds at 2x MIC– 250 μM mansonone G, 62.5 μM Cpd_5, 31.25 μM Cpd_10 and (C) inactive compounds Cpd_8 and Cpd_18 at 250 μM. Scale bar represents 1 μm. (D) Table showing the percentage of nucleoids whose SYTOX intensities are more than 3 times the mean SYTOX intensity of the untreated control. *P*-values are from two-tailed Student’s t-test from pair-wise comparison between each condition and untreated control, n = 3.

Following the findings of mansonone G and its derivatives having antibacterial activity, we were next interested to investigate what the possible mechanism of action of these molecules could be. A previous study done in *E*. *coli* lptD4213 [21] has successfully employed a fluorescent microscopy-based method—BCP—to elucidate the mechanism of action of antibiotics after exposing the bacteria to the antibiotics for two hours. In order to have a better understanding of the mechanism underlying the antibacterial activity of these compounds, we selected *E*. *coli* lptD4213 cells to be treated with the compounds for two hours, for effective comparison with the established data in the previous study [21]. *E*. *coli* lptD4213 cells were incubated with mansonone G for two hours in the presence of fluorescent dyes and then subjected to fluorescent microscopy. The fluorescent dye FM4-64, which stains the cell membrane, is important as it aids in visualizing the cell boundary. FM4-64 staining indicated that mansonone G did not have an effect on the shape of the cell when comparing to the untreated and DMSO-treated controls. Two nucleic acid-staining fluorescent dyes—DAPI and SYTOX Green—were used in this study. DAPI can pass through intact membranes and stain the nucleic acid, thus outlining the position and area of the nucleoid in all cells. The nucleic acid stain SYTOX Green, however, possesses the inability to enter cells with intact membranes, and therefore, was introduced to detect a compromise in the integrity of the bacterial membrane [22]. From the fluorescent images, we were able to determine that after treating the *E*. *coli* cells with mansonone G, SYTOX Green was detected in 85.49% of cells treated with mansonone G (Fig 2B and 2D), indicating membrane compromised cells, whereas for the untreated and DMSO-treated control cells, less than 0.1%. of cells were stained with the dye (Fig 2A and 2D). To rule out the possibility that the membrane compromising effect was specific to only the *E*. *coli* lptD4213 strain used in the study, we performed similar experiments in a wild type *B*. *subtilis* strain and found that 94.42% of mansonone G-treated cells displayed the presence of SYTOX Green (S1A Fig). These finding suggested that mansonone G exhibits its antibacterial activity via a membrane permeabilizing mechanism in both *E*. *coli* lptD4213 and *B*. *subtilis*.

Although the derivatives originated from mansonone G, the addition of functional groups to the backbone of the parent compound created new structures thus making them entirely new entities. Therefore, it was interesting to know how these derivatives, with improved inhibitory activities (lower MIC), compared to the parent molecule in terms of the mechanism by which they exert their activity against the bacteria. From the derivatives screened, we selected compounds **5** and **10** since these were the ones that displayed an improvement in inhibiting the growth of *E*. *coli* lptD4213. Following the incubation of the bacteria cells with each compound for two hours, the results indicated that both the derivatives showed similar morphologies to that of mansonone G (Fig 2B), and SYTOX Green being visible in 100% and 95% of the cells treated with compound **5** and **10**, respectively (Fig 2D). Moreover, SYTOX Green was detectable in less than 1% of cells treated with compounds **8** and **18**, both of which are inactive in *E*. *coli* lptD4213 (Fig 2B and 2D). Thus, these results indicate that, even though derivative compounds 5 and 10 possess better inhibitory activity, they exhibit similar mechanism of action to the parent compound. Collectively, these findings provide some insight on the possible mode by which mansonone G and its derivatives exert their bactericidal activity against the susceptible strains of bacteria.

### Mansonone G displays rapid membrane permeabilizing activity

**Fig 3:**
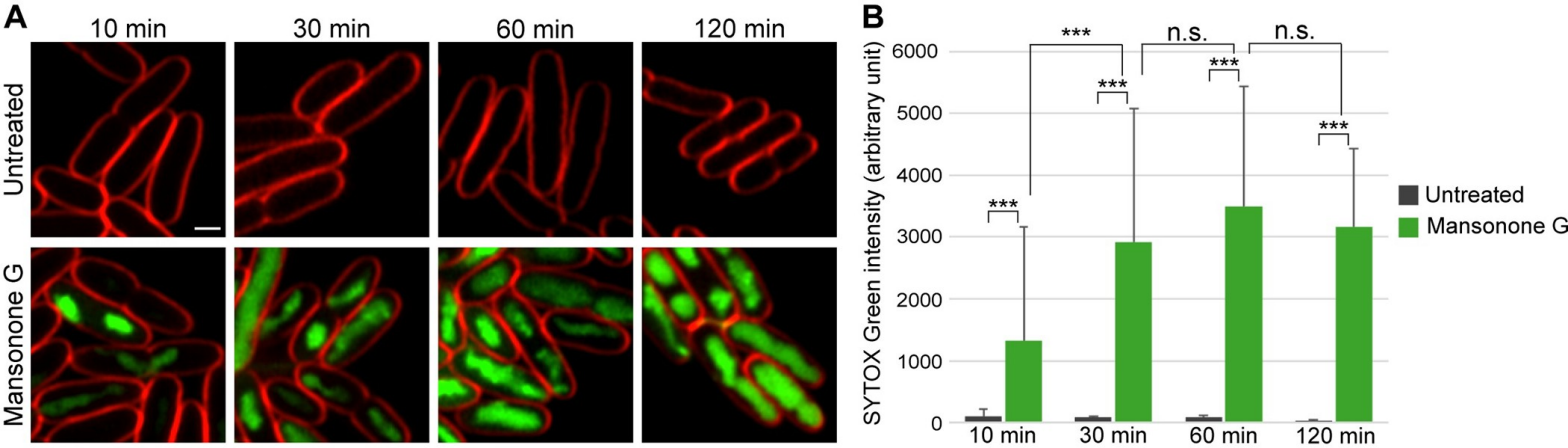
Mansonone G showing rapid membrane permeabilizing activity. *E*. *coli* lptD4213 cells were treated with mansonone G for 10, 30, 60 and 120 minutes, and then stained with FM4-64 (red) and SYTOX Green (green). (A) Upper panels show untreated samples and lower panels show mansonone G treatment. Scale bar represents 1 μm. SYTOX Green intensity has been normalized to that of the brightest sample. (B) A graph showing differences in SYTOX Green intensities between untreated and mansonone G -treated cells at different timepoints. Values represent mean intensities ± STDEV of 45 to 100 individual nucleoids from single cells, per condition. *** *P* < 0.001; two-tailed Student’s t-test, n.s.; not statistically significant.

Since a significantly larger number of cells treated with mansonone G display SYTOX Green signals higher than both the untreated and the DMSO control, at the BCP standard treatment time of two hours, we were interested to see if this activity was present at earlier time points, as membrane permeabilizing action usually occurs at earlier time points [23, 24]. A previous study using BCP found that a membrane-compromising natural product was able to disrupt the *E*. *coli* lptD4213 membrane as early as 10 minutes after exposure with the compound [21]. Hence, we subjected *E*. *coli* lptD4213 cells to fluorescent microscopy after incubating them with mansonone G for 10, 30, 60 and 120 minutes, in the presence of SYTOX Green. The results showed that fluorescent intensity of SYTOX Green in individual cells significantly increased as early as 10 minutes after treatment (Fig 3A and 3B) when compared to the untreated control (*p*-value < 0.001). At 60 minutes, although an upward trend can be seen, this increase was not statistically significant, indicating that at 30 minutes the cells were saturated with SYTOX Green and therefore, further significant increase in fluorescent intensity could not be attained at the later time points. Therefore, these results show that mansonone G is a molecule with rapid membrane permeabilizing activity in a susceptible strain of *E*. *coli*.

## Discussion

In the era of new emerging diseases and alarming rates of antibacterial resistance, coupled with the fact that over the past four to five decades no new classes of antibiotics have been discovered, novel antimicrobials are in dire need [25, 26]. With the lack of success of synthetic antibiotics being introduced to the clinic, in recent years, attention has been turned to natural compounds, of both plant and animal origins, as promising candidates [1]. Therefore, in the quest to find new antibacterial molecules, our study was focused on screening mansonone G, a compound having known to possess a number of medicinal properties [9, 12–14, 17, 18], and its derivatives, hypothesizing the possibility of finding some molecules that are active against Gram-positive and even Gram-negative bacteria.

In the wake of limited discovery of new compounds, medicinal chemists have been shifting their focus to synthesizing derivatives of existing molecules, aiming to enhance their antibacterial activities [27, 28]. Attempts have also been made at modifications of the properties of existing antibiotics to broaden the antibacterial spectrum, with successes seen in second and third generation cephalosporins and broad-spectrum fluoroquinolones [3, 29]. Owing to the high reactivity of the phenolic hydroxyl group of mansonone G, most studies have been inclined to functionally modify this position into ester and ether analogues. In 2016, Hairani *et al* reported that allyl and prenyl ether derivatives of mansonone G possessed antibacterial activities against a number of Gram-positive and one Gram-negative bacteria with marked MIC improvements seen in Gram-positive bacteria especially in *S*. *aureus*, but not in Gram-negative bacteria, when compared to the parent compound [17]. Therefore, here in our study, we wanted to further explore modifications that could expand the antibacterial spectrum of mansonone G against Gram-negative bacteria. However, we found that both mansonone G and some of the derivatives synthesized were active against Gram-positive *S*. *aureus* and *B*. *subtilis* and also against *E*. *coli* lptD4213, with some of the derivatives displaying marked improvements in bactericidal activity. *E*. *coli* lptD4213 is a strain with a mutation on the *lptD* (*imp*) gene [30], and originally isolated from *E*. *coli* MC4100 [31], resulting in a defect in the selective permeability of the outer membrane [32, 33], one of the main defense barriers of Gram-negative bacteria, that lowers the permeability and hinders the entry of antibacterial molecules [20]. Since the compounds in our study were active against *E*. *coli* lptD4213 only and not against wild type *E*. *coli* strain MC4100 and ATCC25922, it is highly possible that the intact LPS-containing outer membrane of wild type *E*. *coli* prevents the compounds from exhibiting their antibacterial activity.

*A*. *baumannii and P*. *aeruginosa* are among the most problematic opportunistic pathogens as they have developed resistance to most of the clinically used antimicrobial agents, and in 2017, the World Health Organization included these two in the list of top priority pathogens [34, 35]. The previous study having shown that mansonone G and its analogues possessed antibacterial activity in Gram-negative *Salmonella typhi* [17], coupled with the urgency to find molecules that are active against the Gram-negative pathogens, we screened mansonone G and all 14 derivatives for antibacterial activity against *A*. *baumannii* ATCC19606, *P*. *aeruginosa* PA01 and a foodborne pathogen *V*. *parahaemolyticus* AHPND, but without successfully obtaining any positive results. Since this study was unable to synthesize any compounds that are active against Gram-negative pathogenic bacteria, further efforts are therefore called for to modify these derivatives to be efficient candidates in penetrating the defense mechanism of Gram-negative bacteria.

BCP has been successful in elucidating the mechanism of action of not only different antibiotic classes, but also members of sub-classes, in many species of bacteria such as *S*. *aureus* [36], *B*. *subtilis* [37], *E*. *coli* lptD4213 [21] and *A*. *baumannii* [38]. In our study, BCP on *E*. *coli* lptD4213 indicated that both the unmodified mansonone G and all of its derivatives, displayed morphologies resembling those of membrane permeabilizing agents. Moreover, mansonone G displayed a similar membrane permeabilizing profile in Gram-positive *B*. *subtilis* (S1 Fig), further reinforcing the conclusion. Membrane permeabilization is one of the successful mechanisms by which molecules exert their antibacterial activities [39, 40]. Various antimicrobial peptides [23, 41, 42], natural product-derived compounds and nanoparticles [43–47] have been shown to exhibit exceptional membrane disrupting activities against the bacteria. Even though concerns have been raised due the low membrane selectivity of the membrane disrupting agents that possibly lead to mammalian cell toxicity [48–51], the compounds still play an valuable role in combating against multi-drug resistant bacteria in various aspects. For example, previous studies have exploited exceptional membrane disrupting activity of a poly-quercetin-based compound in wound dressing [45, 52] and carbonized nanogels/graphene oxide (CNGs/GO) nanocomposite for water filter propose [47].

Membrane disrupting agents can be categorized into many sub-classes, some of which include pore-forming agents like nisin [53], agents such as daptomycin affecting proton gradient [54], polymyxins that bind directly to lipopolysaccharides and molecules like darobactin that bind to outer membrane protein BamA [40], each displaying a distinct morphology on BCP [21]. However, BCP profiles in our study, performed in *E*. *coli* lptD4213, did not exactly match any of these previously elucidated profiles in the same strain [21] and therefore, the exact target, on the membrane, of neither mansonone G nor its derivatives could be identified. As a result, although the presence of SYTOX Green indicates a membrane compromise, suggesting that these compounds possess membrane permeabilizing activities, we were not able to pinpoint the exact macromolecular target. And hence, at present, we can only conclude that in *E*. *coli* lptD4213 and *B*. *subtilis*, mansonone G display morphologies similar to membrane permeabilizing agents, while the exact target has yet to be determined.

Since the exact targets of all the molecules screened in this study could not be identified, it would be interesting to investigate, in future, the targets of mansonone G on the bacterial membrane. However, when identifying the target proteins, it is absolutely crucial that these are real drug interactions and not merely the effects of pan-assay interfering compounds or PAINS [55], artefacts that masquerade drug-like activity leading to false-positive identification. Nevertheless, once identified, the focus should be on modifying the active compounds for better antibacterial activity. Moreover, further research is necessary for synthesizing or modifying the derivatives to be active against Gram-negative bacteria, as the ultimate goal.

## Conclusion

With the threat of MDR bacteria in a continuously upward trend [56], and the process of drug discovery being extremely laborious, resource-consuming, and expensive [57], enhancing the efficiency and spectrum of existing compounds has gained momentum [7]. In this study we have successfully modified mansonone G giving rise to 14 semisynthetic derivatives with enhanced antibacterial activities. Our findings provide a reason for optimism and further backs the fruitfulness of the efforts of synthesizing derivatives of natural compounds as promising candidates for the future of drug discovery.

## Materials and methods

### Extraction of mansonone G from *M*. *gagei* heartwoods

*M*. *gagei* heartwoods was previously purchased at a drug store in Bangkok, Thailand, and after being identified by a taxonomist, was assigned an herbarium number A015376 (BCU) and was stored at the department of Botany, Chulalongkorn University, Bangkok, Thailand. Maceration extraction was carried out in the isolation of mansonone G from ethyl acetate (EtOAc) extract (575 g), as previously described [17]. Briefly, 10 kg of dried, powdered *M*. *gagei* heartwoods was soaked at room temperature in CH_2_Cl_2_ for three days, then filtered and evaporated. A dark-brown CH_2_Cl_2_ extract (276 g, 2.76% yield) was obtained after repeating the process three times. Silica gel quick column (Merck, Germany) was employed to which part of the crude extract was applied. By increasing the polarity with EtOAc, a stepwise elution was performed with hexane, giving rise to 8 fractions of mansonones. Mansonone G was isolated from fractions 4 and 5 as an orange powder, its structure identified by ^1^H NMR analysis and compared to previously reported data [58] to further verify the compound.

### Synthesis of mansonone G derivatives

Mansonone G (8.0 g) was utilized as the starting material for further functional modifications. Fig 1 shows the preparation of **substrate A** [17] and compounds **5–16** involving two different procedures. **Substrate A** and compounds **5–14** were synthesized according to Hairani’s method, with a few tweaks aiming to increase yields and shorten reaction times [17]. Specifically, The Williamson etherification was conducted in a rather forcing condition, in which DMF was employed as solvent and the temperature was up to 100°C, resulting in good-to-excellent yields (57–96%) of obtained products, and predictably, shorter reaction times (5–30 minutes). Interestingly, in the etherification with 1,3-dibromopropane, the further substitution of one more mansonone G molecule to the newly formed product **8** led to the formation of a coupling product **14**.

To broaden the scope of the analog, the alkylation of mansonone G with epichlorohydrin was carried out. After being refluxed for 2 hours, the epoxide **15** (16% yield) along with the ring-opening product **16** (37% yield) were obtained [17].

Compound **18** was attained by a two-step modification of **substrate A**. At first, **substrate A** underwent the epoxidation employing *m*-CPBA in CHCl_3_ for 14 hours to afford the epoxide **17** in good yield (68%). Subsequently, 8% perchloric acid was used to hydrolyze the epoxide resulting in the diol **18** (21% yield) [59, 60].

All derivatives were characterized by ^1^H, ^13^C NMR and MS spectrometric analysis, whereby analogues **5** and **8–18** were identified as new semisynthetic derivatives of mansonone G. All reagent grade chemicals and solvents were purchased from standard suppliers and were used as received without further purification. ^1^H and ^13^C NMR spectra were recorded in a suitable deuterated solvent on JEOL JNM-ECZ500R/S1 operating at 500 MHz (^1^H) and 126 MHz (^13^C). NMR spectra were further processed using the Mnova software, version 14.2.2 (Mestrelab Research, S.L.). ^1^H and ^13^C NMR spectra data can be found in S1 File. High-resolution mass spectroscopy was performed on JEOL SpiralTOF JMS-S3000 MALDI Imaging-TOF/TOF Mass Spectrometer at the Scientific and Technological Research Equipment Centre (STREC) of Chulalongkorn University, Bangkok, Thailand.

Compounds **5**–**16** were prepared according to the following procedure. A dark color mixture of mansonone G (200.0 mg, 0.8 mmol) and K_2_CO_3_ (228.0 mg, 1.6 mmol) in 8 mL of DMF was stirred at room temperature for 15 minutes. Then alkyl bromide (3.2 mmol) was added, and the reaction was stirred at 110°C. At the point the color of the reaction changed to orange and the completion of the reaction was confirmed by TLC (0.5–3 h), water was added to the reaction mixture and the aqueous layer was extracted with EtOAc (40 mL, two times). The organic layer was separated and dried over anhydrous Na_2_SO_4_. The product as orange powder was purified by chromatography (No.7734, Merck) using hexane: EtOAc (4:1).

To a two-neck round-bottomed flask with 8 mL of epichlorohydrin was added mansonone G (244 mg, 1 mmol) while stirring. Then, the mixture was refluxed for 10 minutes and an ethanolic solution of NaOH (10 mg, 0.25 mmol) in 95% EtOH (1 mL) was added dropwise. The progress of the reaction was monitored by TLC. After 2 h, the reaction mixture was cooled at room temperature, added water, and extracted with EtOAc (15 mL, three times). The combined organic layer was dried over anhydrous Na_2_SO_4_, concentrated. **15** and **16** were obtained as orange powder on silica gel column (No.7734, Merck) using hexane-CH_2_Cl_2_-EtOAc 6:2:3.

To a stirred solution of *m*-CPBA (0.84 g, 4.9 mmol) in CHCl_3_ (277 mL) held at 0°C was added a solution of **substrate A** (1.21 g, 3.9 mmol) dissolved in CHCl_3_ (100 mL) over the course of 30 minutes. After being stirred overnight (14 hours), the mixture was washed with 10% NaHCO_3_ solution and the resulting organic layer was dried over anhydrous Na_2_SO_4_. The solvent was concentrated *in vacuo* and the residue was purified by chromatography on silica gel (No.7734, Merck) using a mixture of CH_2_Cl_2_-EtOAc-acetone 40:10:2 to afford the epoxide **17** as orange powder.

To a solution of epoxide **17** (216 g, 0.65 mmol) in 6 mL of THF was added 1.15 mL of water. The solution was stirred and 0.2 mL of 8% HClO_4_ was added. After stirring for 24 hours under N_2_ at room temperature, 15 mL of brine was added, and the mixture was extracted several times with EtOAc. The organic phase was washed with dilute NaHCO_3_ and brine, dried over Na_2_SO_4_, evaporated under reduced pressure, and purified by chromatography on silica gel (No.7734, Merck) using hexane-EtOAc-acetone mixture 4:2:1 to afford the diol **18** as orange powder.

5-isopropyl-3,8-dimethyl-6-((3-methylbut-2-en-1-yl)oxy)naphthalene-1,2-dione (**substrate A**, 223.6 mg, 87.5%). ^1^H NMR (400 MHz, CDCl_3_): *d* 7.70 (s, 1H), 6.59 (s, 1H), 5.48 (m, 1H), 4.59 (d, *J =* 5.2 Hz), 3.58 (m, 1H), 2.61 (s, 3H), 2.04 (s, 3H), 1.81 (s, 3H), 1.75 (s, 3H), 1.36 (d, *J =* 6.8 Hz, 6H).

5-isopropyl-3,8-dimethyl-6-(prop-2-yn-1-yloxy)naphthalene-1,2-dione (**5**, 202.4 mg, 87.5%). ^1^H NMR (400 MHz, CDCl_3_): *d* 7.74 (s, 1H), 6.73 (s, 1H), 4.82 (s, 2H), 3.61 (m, 1H), 2.67 (s, 3H), 2.60 (s, 1H), 2.08 (s, 3H), 1.41 (d, *J =* 7.1 Hz, 6H). ^13^C NMR (100 MHz, CDCl_3_): *d* 182.4, 180.7, 161.0, 146.1, 138.3, 135.3, 134.8, 134.8, 123.7, 116.3, 77.4, 76.4, 55.9, 26.8, 23.7, 21.4, 16.1. HRMS (ESI) calcd for C_18_H_18_O_3_ [M+Na]^+^: 305.1256, found 305.1141.

6-(cinnamyloxy)-5-isopropyl-3,8-dimethylnaphthalene-1,2-dione (**6**, 204 mg, 68.9%). ^1^H NMR (400 MHz, CDCl_3_): *d* 7.72 (s, 1H), 7.37 (m, 5H), 6.66 (s, 1H), 6.76 (m, 2H), 6.42 (m, 1H), 3.63 (m, 1H), 2.64 (s, 3H), 2.06 (s, 3H), 1.42 (d, *J* = 7.0 Hz, 6H).

6-(benzyloxy)-5-isopropyl-3,8-dimethylnaphthalene-1,2-dione (**7**, 235.2 mg, 85.9%). ^1^H NMR (400 MHz, CDCl_3_): *d* 7.72 (s, 1H), 7.42 (m, 5H), 6.70 (s, 1H), 5.16 (s, 2H), 3.63 (m, 1H), 2.62, (s, 3H), 2.06 (s, 3H), 1.38 (d, *J* = 7.1 Hz, 6H).

6-(3-bromopropoxy)-5-isopropyl-3,8-dimethylnaphthalene-1,2-dione (**8**, 224 mg, 74.9%). ^1^H NMR (400 MHz, CDCl_3_): *d* 7.72 (s, 1H), 6.66 (s, 1H), 4.25 (t, *J =* 5.8 Hz, 2H), 3.65 (t, *J =* 5.8 Hz, 2H), 3.62 (m, 1H), 2.65 (s, 3H), 2.43 (m, 2H), 2.08 (s, 3H), 1.40 (d, *J =* 7.0 Hz, 6H). ^13^C NMR (100 MHz, CDCl_3_): *d* 182.4, 180.6, 162.2, 146.4, 138.3, 135.2, 134.7, 134.1, 123.2, 115.6, 66.0, 32.0, 29.5, 26.8, 23.7, 21.6, 16.1. HRMS (ESI) calcd for C_18_H_21_BrO_3_ [M+Na]^+^: 387.0674, found 387.0569.

6-(4-bromobutoxy)-5-isopropyl-3,8-dimethylnaphthalene-1,2-dione (**9**, 266.4 mg, 85.8%). ^1^H NMR (400 MHz, CDCl_3_): *d* 7.73 (s, 1H), 6.62 (s, 1H), 4.12 (t, *J =* 5.9 Hz, 2H), 3.62 (m, 1H), 3.53 (t, *J =* 5.9 Hz, 2H), 2.65 (s, 3H), 2.04–2.15 (m, 4H), 2.08 (s, 3H), 1.41 (d, *J =* 7.0 Hz, 6H). ^13^C NMR (100 MHz, CDCl_3_): *d* 182.5, 180.6, 162.4, 146.5, 138.3, 135.2, 134.6, 134.2, 123.1, 115.5, 67.5, 32.9, 27.7, 26.9, 23.7, 21.5, 16.1. HRMS (ESI) calcd for C_19_H_23_BrO_3_ [M+Na]^+^: 401.0831, found 401.0736.

6-((2-(bromomethyl)benzyl)oxy)-5-isopropyl-3,8-dimethylnaphthalene-1,2-dione (**10**, 200 mg, 57.1%). ^1^H NMR (400 MHz, CDCl_3_): *d* 7.75 (s, 1H), 7.50 (s, 1H), 7.38–7.42 (m, 3H), 6.71 (s, 1H), 5.18 (s, 2H), 4.54 (s, 2H), 3.66 (m, 1H), 2.64 (s, 3H), 2.08 (s, 3H), 1.41 (d, *J =* 7.0 Hz, 6H). ^13^C NMR (100 MHz, CDCl_3_): *d* 182.5, 180.6, 162.1, 146.4, 138.4, 138.3, 136.6, 135.2, 134.8, 134.6, 129.3, 128.9, 128.2, 127.5, 123.3, 116.0, 70.2, 33.0, 26.9, 23.7, 21.5, 16.1. HRMS (ESI) calcd for C_23_H_23_BrO_3_ [M+Na]^+^: 449.0831, found 449.0735.

6-((3-(bromomethyl)benzyl)oxy)-5-isopropyl-3,8-dimethylnaphthalene-1,2-dione (**11**, 236.4 mg, 67.5%). ^1^H NMR (400 MHz, CDCl_3_): *d* 7.75 (s, 1H), 7.50 (s, 1H), 7.38–7.42 (m, 3H), 6.71 (s, 1H), 5.18 (s, 2H), 4.54 (s, 2H), 3.66 (m, 1H), 2.64 (s, 3H), 2.08 (s, 3H), 1.41 (d, *J =* 7.0 Hz, 6H). ^13^C NMR (100 MHz, CDCl_3_): *d* 182.5, 180.6, 162.1, 146.4, 138.4, 138.3, 136.6, 135.2, 134.8, 134.6, 129.3, 128.9, 128.2, 127.5, 123.3, 116.0, 70.2, 33.0, 26.9, 23.7, 21.5, 16.1. HRMS (ESI) calcd for C_23_H_23_BrO_3_ [M+Na]^+^: 449.0831, found 449.0735.

6-((4-(bromomethyl)benzyl)oxy)-5-isopropyl-3,8-dimethylnaphthalene-1,2-dione (**12**, 252 mg, 72%). ^1^H NMR (400 MHz, CDCl_3_): *d* 7.65 (s, 1H), 7.38 (d, *J =* 8.2 Hz, 2H), 7.34 (d, *J =* 8.2 Hz, 2H), 6.61 (s, 1H), 5.08 (s, 2H), 4.45 (s, 2H), 3.56 (m, 1H), 2.55 (s, 3H), 1.99 (s, 3H), 1.31 (d, *J =* 7.1 Hz, 6H). ^13^C NMR (100 MHz, CDCl_3_): *d* 182.5, l80.6, 162.1, 146.4, 138.3, 138.0, 136.1, 135.2, 134.8, 134.5, 129.5, 127.9, 123.3, 116.0, 70.2, 32.9, 26.9, 23.7, 21.5, 16.1. HRMS (ESI) calcd for C_23_H_23_BrO_3_ [M+Na]^+^: 449.0831, found 449.0748.

6-(2-hydroxyethoxy)-5-isopropyl-3,8-dimethylnaphthalene-1,2-dione (**13**, 200 mg, 84.7%). ^1^H NMR (400 MHz, CDCl_3_): *d* 7.73 (s, 1H), 6.64 (s, 1H), 4.21 (t, *J =* 4.4 Hz, 2H), 4.09 (t, *J =* 4.4 Hz, 2H), 3.62 (m, 1H), 2.63 (s, 3H), 2.07 (s, 3H), 1.41 (d, *J* = 7.0 Hz, 6H). ^13^C NMR (100 MHz, CDCl_3_): *d* 182.5, 180.5, 162.4, 146.5, 138.4, 135.2, 134.6, 134.4, 123.2, 115.8, 69.8, 61.2, 27.0, 23.7, 21.6, 16.1. HRMS (ESI) calcd for C_17_H_20_O_4_ [M+Na]^+^: 311.1362, found 311.1267.

6,6’-(propane-1,3-diylbis(oxy))bis(5-isopropyl-3,8-dimethylnaphthalene-1,2-dione) (**14**, 102.4 mg, 23.6%). ^1^H NMR (400 MHz, CDCl_3_): *d* 7.69 (s, 2H), 6.63 (s, 2H), 4.31 (t, *J* = 6.0 Hz, 4H), 3.60 (m, 2H), 2.60 (s, 6H), 2.44 (m, 2H), 2.03 (s, 6H), 1.37 (d, *J* = 7.0 Hz, 12H). ^13^C NMR (100 MHz, CDCl_3_): *d* 182.4, 180.5, 162.2, 146.4, 138.3, 135.3, 134.7, 134.2, 123.2, 115.5, 65.0, 28.9, 26.8, 23.7, 21.5, 16.1. HRMS (ESI) calcd for C_33_H_36_O_6_ [M+Na]^+^: 551.2512, found 551.2436.

5-isopropyl-3,8-dimethyl-6-(oxiran-2-ylmethoxy)naphthalene-1,2-dione (**15**, 48.0 mg, 16%). ^1^H NMR (400 MHz, CDCl_3_): *d* 7.73 (s, 1H), 6.62 (s, 1H), 4.40 (dd, *J* = 10.8, 2.4 Hz, 1H), 4.02 (dd, *J* = 11.2, 6.4 Hz, 1H), 3.63 (m, 1H), 3.44 (m, 1H), 2.99 (t, *J* = 4.3 Hz, 1H), 2.80 (dd, *J* = 4.7, 2.6 Hz, 1H), 2.64 (s, 3H), 2.06 (s, 3H), 1.43 (dd, *J* = 6.8, 3.2 Hz, 6H).

6-(3-chloro-2-hydroxypropoxy)-5-isopropyl-3,8-dimethylnaphthalene-1,2-dione (**16**, 125 mg, 37.1%). ^1^H NMR (400 MHz, CDCl_3_): *d* 7.71 (s, 1H), 6.66 (s, 1H), 4.39 (m, 1H), 4.24 (d, *J =* 5.2 Hz, 2H), 3.86 (dd, *J =* 11.3, 5.1 Hz, 1H), 3.81 (dd, *J =* 11.3, 5.4 Hz, 1H), 3.60 (m, 1H), 2.59 (s, 3H), 2.04 (s, 3H), 1.38 (d, *J =* 7.0 Hz, 6H).

6-(3,3-dimethyloxiran-2-yl)methoxy)-5-isopropyl-3,8-dimethylnaphthalene-1,2-dione (**17**, 856 mg, 67.5%). ^1^H NMR (400 MHz, CDCl_3_): *d* 7.64 (s, 1H), 6.54 (s, 1H), 4.21 (dd, *J =* 10.8, 4.2 Hz, 1H), 4.05 (dd, *J =* 10.8, 6.3 Hz, 1H), 3.54 (m, 1H), 3.13 (dd, *J =* 6.1, 4.3 Hz, 1H), 2.57 (s, 3H), 1.99 (s, 3H), 1.36 (s, 3H), 1.35 (d, *J =* 4.8 Hz, 3H), 1.34 (d, *J =* 4.8 Hz), 1.33 (s, 3H). ^13^C NMR (100 MHz, CDCl_3_): *d* 182.5, 180.6, 162.4, 146.4, 138.2, 135.3, 134.7, 134.4, 123.4, 115.8, 67.7, 60.8, 58.2, 27.1, 24.5, 23.7, 21.4, 19.0, 16.1. HRMS (ESI) calcd for C_20_H_24_O_4_ [M+Na]^+^: 351.1675, found 351.1597.

6-(2,3-dihydroxy-3-methylbutoxy)-5-isopropyl-3,8-dimethylnaphthalene-1,2-dione (**18**, 46.5 mg, 20.5%). ^1^H NMR (400 MHz, DMSO-*d*_*6*_): *d* 7.91 (s, 1H), 6.92 (s, 1H), 5.02 (br, -OH), 4.48 (br, -OH), 4.36 (d, *J =* 9.9 Hz, 1H), 3.94 (t, *J =* 8.8 Hz, 1H), 3.70 (m, 1H), 3.64 (m, 1H), 2.55 (s, 3H), 1.97 (s, 3H), 1.38 (d, *J =* 7.1 Hz, 3H), l.36 (d, *J =* 7.0 Hz, 3H), 1.18 (s, 3H), 1.12 (s, 3H). ^13^C NMR (100 MHz, DMSO-*d*_*6*_): *d* 182.1, 180.0, 162.8, 145.3, 138.0, 134.7, 134.0, 133.9, 122.3, 115.9, 76.0, 70.9, 70.7, 27.5, 26.2, 24.2, 22.9, 21.1, 15.3. HRMS (ESI) calcd for C_20_H_26_O_5_ [M+Na]^+^: 369.1780, found 369.16.

### Bacteria strains and compounds

*Bacillus subtilis* PY79, *Staphylococcus aureus* ATCC29213, *Escherichia coli* lptD4213, *Escherichia coli* MC4100, *Escherichia coli* ATCC25922, *Acinetobacter baumannii* ATCC19606, *Pseudomonas aeruginosa* PA01 and *Vibrio papahaemolyticus* AHPND were used in this study.

Mansonone G and its derivatives are listed in Table 1. All compounds used are dissolved in DMSO as stocks having a concentration of 100 mM.

### Minimal inhibitory concentration

The minimal inhibitory concentration (MIC) for each compound was determined by microdilution method as previously described [38]. Briefly, single colonies of bacteria were grown in Luria-Bertani medium (LB) overnight at 30°C on a roller at 50 rpm. Overnight cultures were diluted 100-fold and further incubated at 30°C on a roller at 50 rpm until exponential phase, where OD_600_ of 0.2 was attained. The culture was then diluted further in LB, to obtain 200,000 cells/ml, and aspirated into individual wells of 96-well plate that previously contained a two-fold serial dilution of each compound in LB. A positive growth control containing bacteria in LB without compounds but with DMSO in equal volumes as that of each compound and a negative media control of LB alone, were included for every set of serially diluted compounds. The plate was incubated at 30°C for 24 hours. MIC was recorded the following day by visually observing each well of the plate, in comparison with both the positive and the negative growth controls and determined as the lowest concentration of each compound in which no visible growth of bacteria could be observed. MIC determination was performed in triplicates, for every strain of bacteria tested and reported as the mode value from these three independent experiments (S2 Table).

### Fluorescent microscopy

Single colonies of *E*. *coli* lptD4213, grown overnight at 30°C on a roller at 50 rpm, were diluted 100-fold and allowed to grow at 30°C on a roller at 50 rpm until exponential phase, where OD_600_ of 0.2 was obtained. For experiments on *B*. *subtilis*, single colonies from fleshly streaked plates of *B*. *subtilis* were inoculated into LB medium and incubated at 30°C on a roller at 50 rpm until exponential phase.

Compounds were added at 2 times the MIC for all experiments. The cultures were further incubated, on the roller at 30°C for 120 minutes. Each condition was accompanied by a control of untreated culture and a solvent control of DMSO at 0.25% v/v for *E*. *coli* lptD4213 and 0.125% v/v for *B*. *subtilis*, which corresponds to the maximum amount of DMSO present in the compound-treated bacterial cultures subjected to fluorescent microscopy. For the experiment observing membrane disruption at different time points, incubation with mansonone G was for 10, 30, 60 and 120 minutes.

Three fluorescent dyes were used in all of fluorescent microscopy work and were obtained from Invitrogen, USA. FM4-64 and SYTOX Green were dissolved in DMSO at concentrations of 1 mg/ml and 5 mM respectively, while DAPI was dissolved in distilled water at a stock concentration of 2 mg/ml. For fluorescent microscopy in *E*. *coli* lptD4213, 2 μg/ml FM4-64, 4 μg/ml DAPI and 0.5 μM SYTOX Green were used whereas in *B*. *subtilis*, 1 μg/ml FM4-64, 1 μg/ml DAPI and 0.5 μM SYTOX Green were used. All samples—both controls and compound-treated—contain the same number and concentration of fluorescent dyes. After adding fluorescent dyes, the samples were incubated for a further 5 minutes. The cultures were then concentrated to 1/10 the original volume, after which 3 microliters was loaded to a concave glass slide that contained an agarose pad (1.2% agarose in 20% LB) on it. Fluorescent microscopy was performed on ZEISS LSM 800 with Airyscan, using laser intensities of 2% for FM4-64 (excitation 506 nm/ emission 751 nm), 5% for DAPI (excitation 353 nm/ emission 465 nm) and 0.5% for SYTOX Green (excitation 504 nm/ emission 524 nm), with consistent imaging parameters in all experiments.

### Image analysis

All images obtained from fluorescent microscopy were first preprocessed on Fiji software [61] and then analyzed on CellProfiler 4.2.1 software [62]. Examples of whole-field images are shown in S2 and S3 Figs. From the cell parameters generated, the nucleoid outline was used to define the area in which to measure the SYTOX Green intensity, after which the background intensity of the corresponding cell was subtracted, in order to obtain the intensity of SYTOX Green for each individual cell. SYTOX Green intensity was measured from the unadjusted image obtained from fluorescent microscopy.

### Statistical analysis

All statistical analysis was performed on Microsoft Excel version 16.59. Statistical analysis of the percent of cells stained with SYTOX Green was from 3 separate images, with nucleoid outlines ranging from 100 to 500 per image for Fig 2D and S1 Table, and 40 to 300 for S1B Fig, for all treatment conditions. The percent of cells stained with SYTOX Green is the percent of cells, per image, with SYTOX Green intensities more than 3 times the mean SYTOX intensity of the untreated control and has been presented as percent ± standard deviation (STDEV) in Fig 2D and S1 Table for *E*. *coli* lptD4213 and S1B Fig for *B*. *subtilis*. Two-tailed Student’s t-test was performed for all treatment conditions in comparison to the untreated control.

Statistical analysis of SYTOX Green intensities at different time points was from 45 to 100 individual cells per time point. Means of SYTOX Green intensities of untreated and mansonone G-treated cells for each time point were plotted in Microsoft Excel. Two-tailed Student’s t-test was performed for all pairs of time points of mansonone G-treated cells and also for untreated and mansonone G-treated cells at each time point (Fig 3B). Minimal raw data for Fig 3B can be found in S3 Table.

## Supporting information

S1 Fig Mansonone G showing membrane permeabilizing activity in *B*. *subtilis* PY79. (PDF)

S2 Fig Whole-field examples of mansonone G and its derivatives showing membrane permeabilizing activity in *E*. *coli* lptD4213. (PDF)

S3 Fig Whole-field examples of mansonone G showing membrane permeabilizing activity in *B*. *subtilis* PY79. (PDF)

S1 Table The percentage of nucleoids with SYTOX intensities more than 3 times the mean SYTOX intensity of the untreated control, in *E*. *coli* lptD4213 cells. (XLSX)

S2 Table Minimal inhibitory concentration of mansonone G and its derivatives performed in triplicate, in eight bacterial strains. (XLSX)

S3 Table SYTOX Green intensities of individual cells from untreated and mansonone G-treated conditions, at different time points in *E*. *coli* lptD4213 cells. (XLSX)

S1 File NMR of substrate A and compounds 5–18. (PDF)
