# Supplementary material for: Mansonone G and its derivatives exhibit membrane permeabilizing activities against bacteria
Source: PLoS One. 2022 Sep 1;17(9):e0273614. doi: 10.1371/journal.pone.0273614 (PMC9436067; doi:10.1371/journal.pone.0273614)
Supplement: S1 Fig — (PDF) [file pone.0273614.s001.pdf]

A

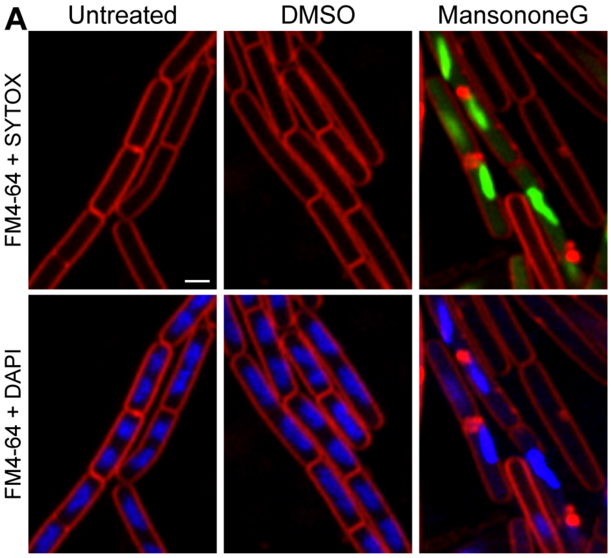

B

Treatment conditions	No. of nucleoids	% nucleoids stained with SYTOX green	*P*-values
Untreated_1	238	0	
Untreated_2	322	0	
Untreated_3	248	0.40	
DMSO_1	273	0	
DMSO_2	253	0	0.423
DMSO_3	276	0	
Mansonone G_1	76	92.11	
Mansonone G_2	44	95.45	< 0.001
Mansonone G_3	70	95.71	

S1 Fig. Mansonone G showing membrane permeabilizing activity in *B. subtilis* PY79.

(A) *B. subtilis* PY79 cells were treated for 120 minutes with mansonone G and then stained with 1 µg/ml FM4-64 (red), 1 µg/ml DAPI (blue) and 0.5 µM SYTOX Green (Green). Upper panels show FM4-64 and SYTOX Green while lower panels show FM4-64 and DAPI, for each treatment condition; untreated control, 0.125% v/v DMSO treatment and mansonone G treatment at 31.25 µM (2x MIC). Scale bar represents 1 µm. (B) Table showing the percentage of nucleoids whose SYTOX intensities are more than 3 times the mean SYTOX intensity of the untreated control. P-values are from two-tailed Student's t-test, n = 3.
