# Supplementary material for: Mansonone G and its derivatives exhibit membrane permeabilizing activities against bacteria
Source: PLoS One. 2022 Sep 1;17(9):e0273614. doi: 10.1371/journal.pone.0273614 (PMC9436067; doi:10.1371/journal.pone.0273614)
Supplement: S2 Fig — (PDF) [file pone.0273614.s002.pdf]

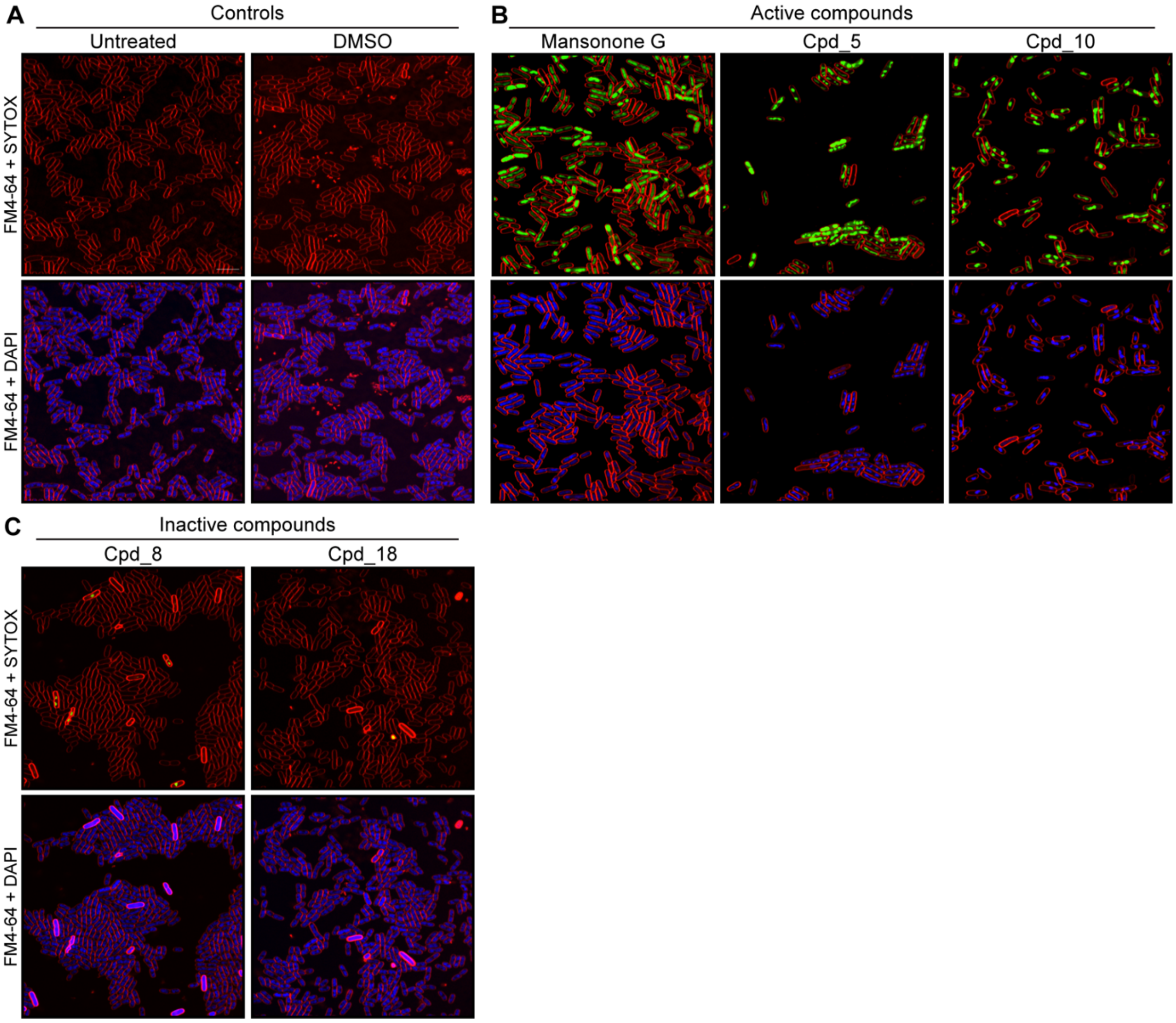

S2 Fig. Whole-field examples of mansonone G and its derivatives showing membrane permeabilizing activity in *E. coli* lptD4213.

E. coli lptD4213 cells were treated for 120 minutes with compounds and then stained with 2 μg/ml FM4-64 (red), 4 μg/ml DAPI (blue) and 0.5 μM SYTOX Green (Green). Upper panels show FM4-64 and SYTOX Green while lower panels show FM4-64 and DAPI, for each treatment condition; (A) untreated control and 0.25% v/v DMSO-treated control (B) active compounds at 2x MIC – 250 μM mansonone G, 62.5 μM Cpd_5, 31.25 μM Cpd_10, (C) inactive compounds at 250 μM Cpd_8 and Cpd_18. Scale bar represents 5 μm.
