# Supplementary material for: Mansonone G and its derivatives exhibit membrane permeabilizing activities against bacteria
Source: PLoS One. 2022 Sep 1;17(9):e0273614. doi: 10.1371/journal.pone.0273614 (PMC9436067; doi:10.1371/journal.pone.0273614)
Supplement: S3 Fig — (PDF) [file pone.0273614.s003.pdf]

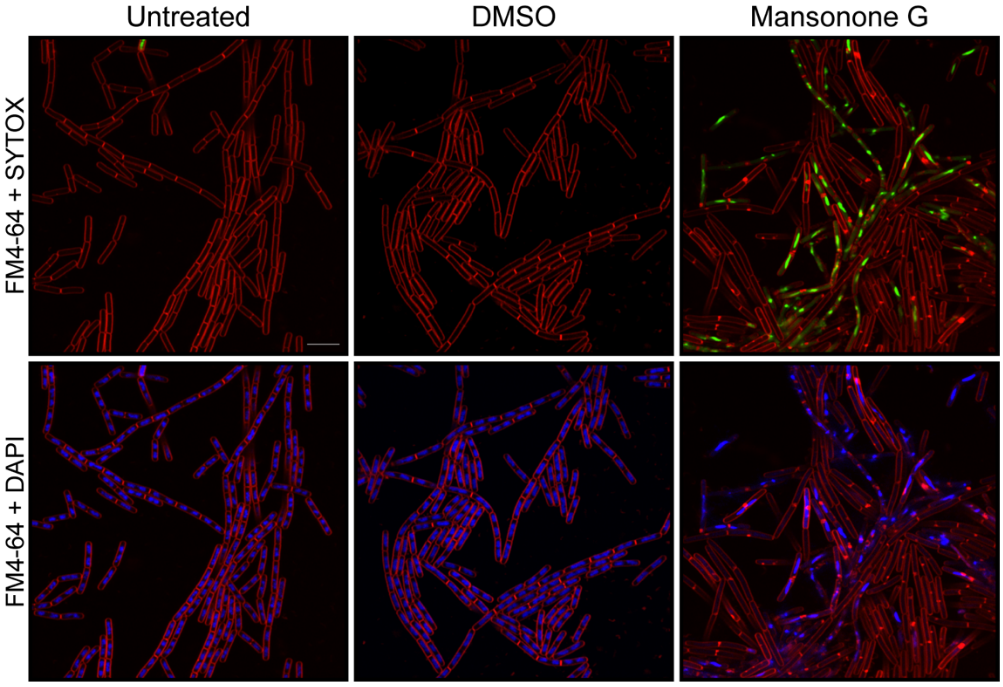

S3 Fig. Whole-field examples of mansonone G showing membrane permeabilizing activity in *B. subtilis* PY79.

B. subtilis PY79 cells were treated for 120 minutes with mansonone G and then stained with 1 μg/ml FM4-64 (red), 1 μg/ml DAPI (blue) and 0.5 μM SYTOX Green (Green). Upper panels show FM4-64 and SYTOX Green while lower panels show FM4-64 and DAPI, for each treatment condition; untreated control, 0.125% v/v DMSO treatment and mansonone G treatment at 31.25 μM (2x MIC). Scale bar represents 5 μm.
