# Supplementary material for: Mansonone G and its derivatives exhibit membrane permeabilizing activities against bacteria
Source: PLoS One. 2022 Sep 1;17(9):e0273614. doi: 10.1371/journal.pone.0273614 (PMC9436067; doi:10.1371/journal.pone.0273614)
Supplement: S1 File — (PDF) [file pone.0273614.s007.pdf]

S1 File. NMR of substrate A and compounds 5–18.

A. Substrate A.

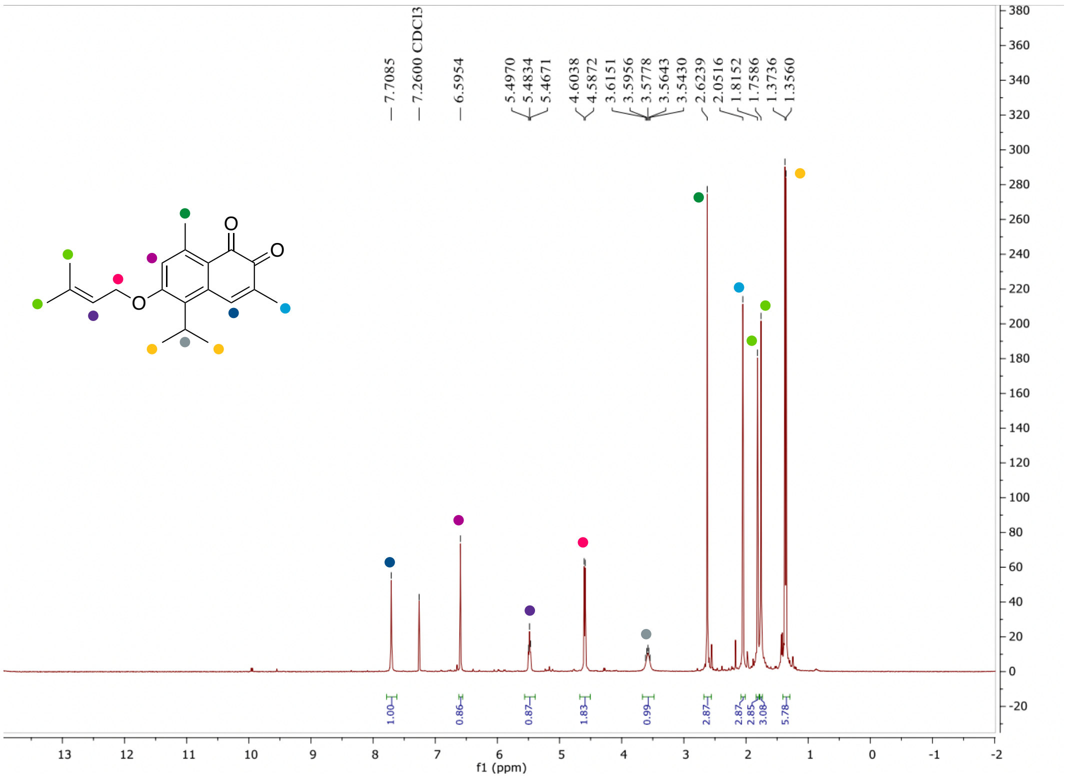

^{1}H NMR of 5-isopropyl-3,8-dimethyl-6-((3-methylbut-2-en-1-yl)oxy)naphthalene-1,2-dione (**Substrate A**)

B. Compound 5.

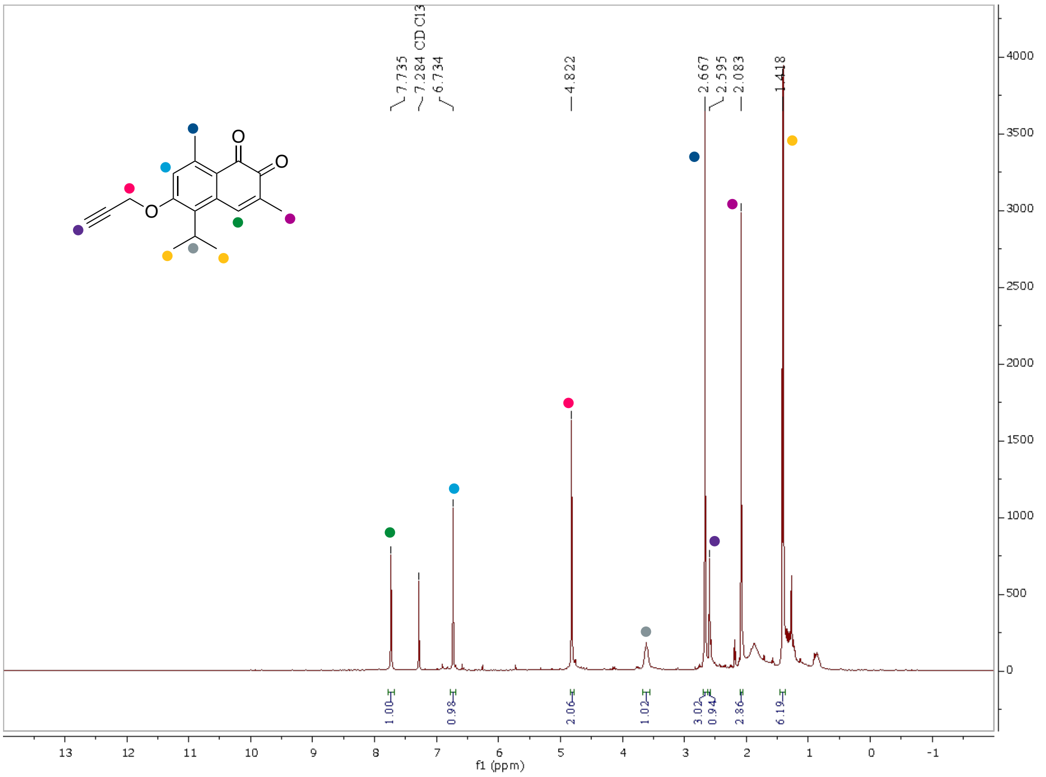

^{1}H NMR of 5-isopropyl-3,8-dimethyl-6-(prop-2-yn-1-yloxy)naphthalene-1,2-dione (**5**)

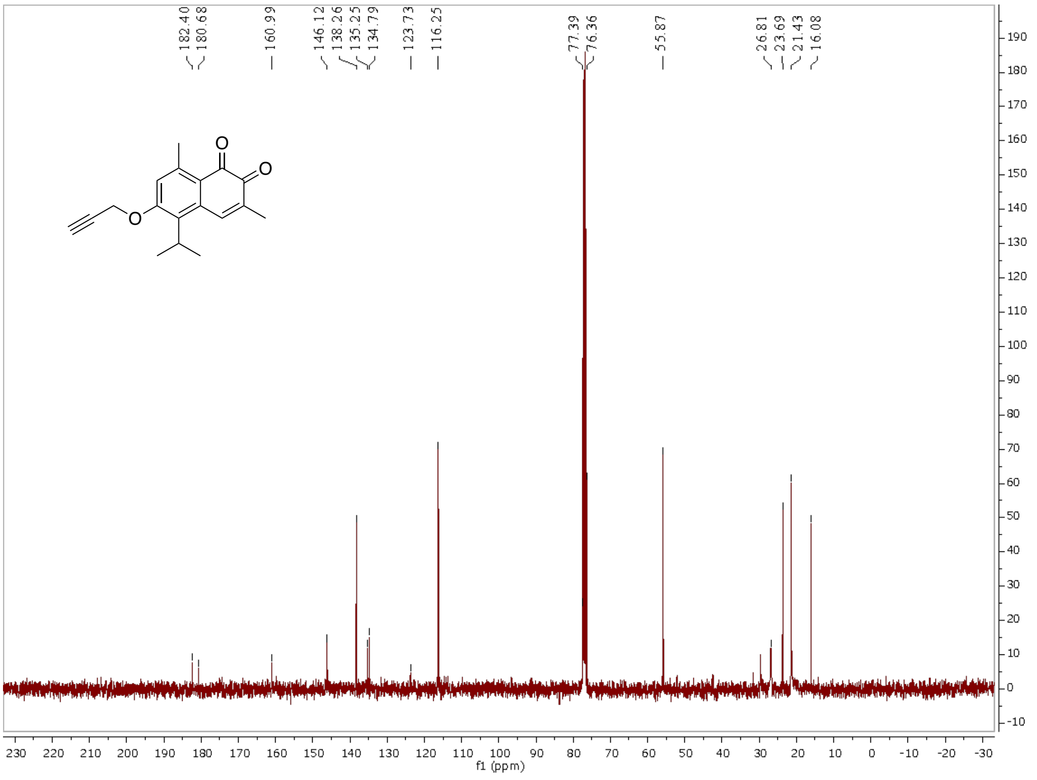

^{13}C NMR of 5-isopropyl-3,8-dimethyl-6-(prop-2-yn-1-yloxy)naphthalene-1,2-dione (**5**)

C. Compound 6.

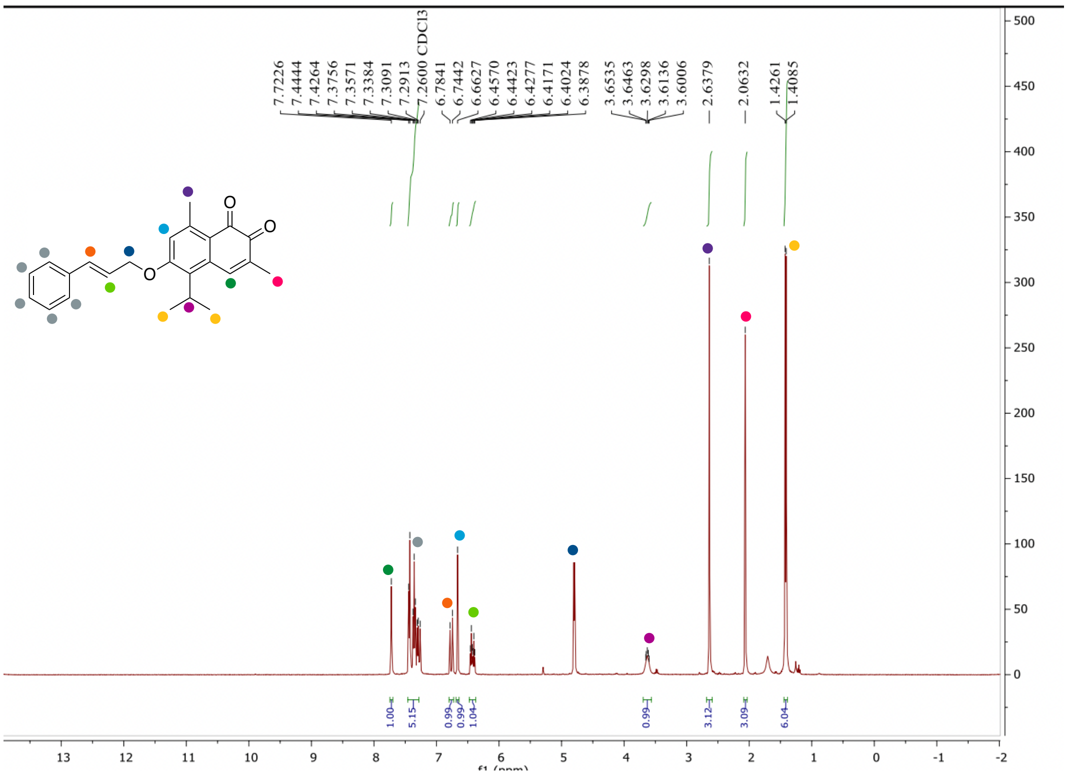

^{1}H NMR of 6-(cinnamyloxy)-5-isopropyl-3,8-dimethylnaphthalene-1,2-dione (**6**)

D. Compound 7.

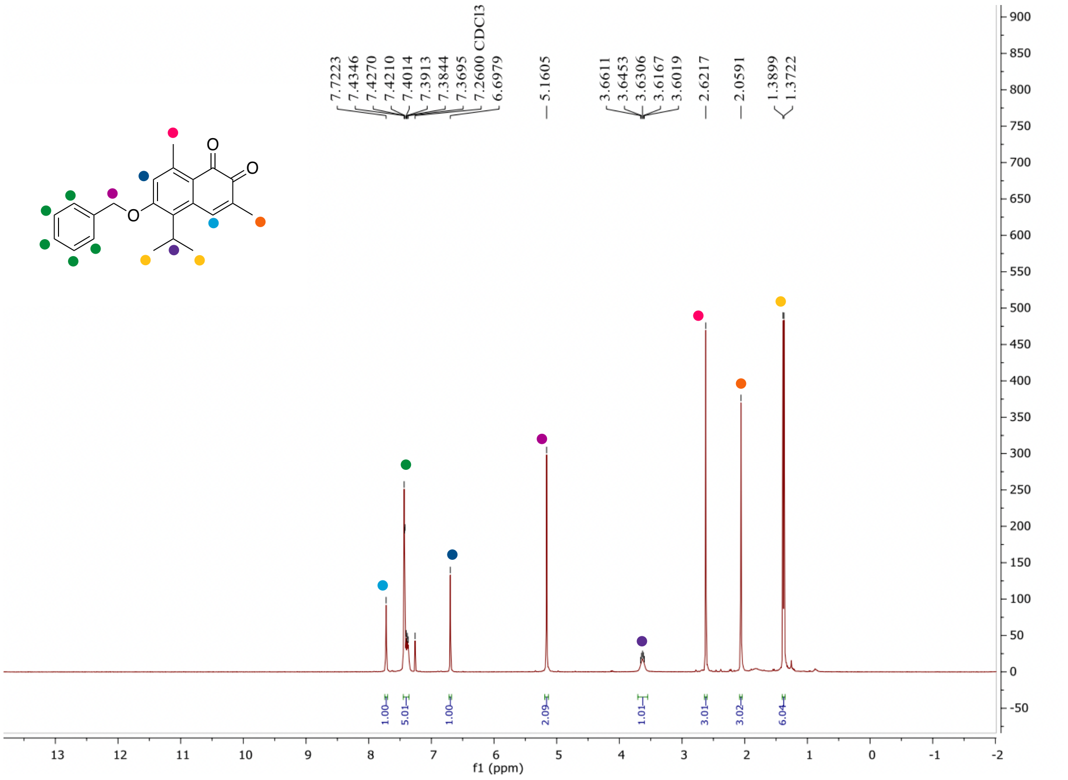

^{1}H NMR of 6-(benzyloxy)-5-isopropyl-3,8-dimethylnaphthalene-1,2-dione (**7**)

E. Compound 8.

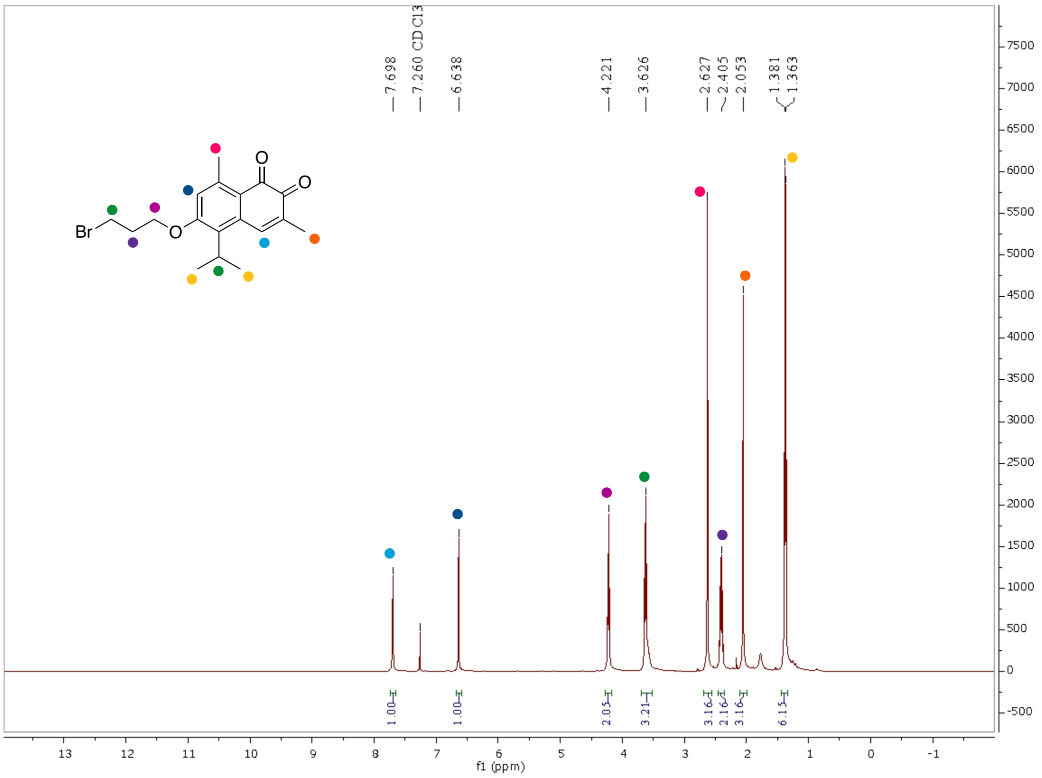

^{1}H NMR of 6-(3-bromopropoxy)-5-isopropyl-3,8-dimethylnaphthalene-1,2-dione (**8**)

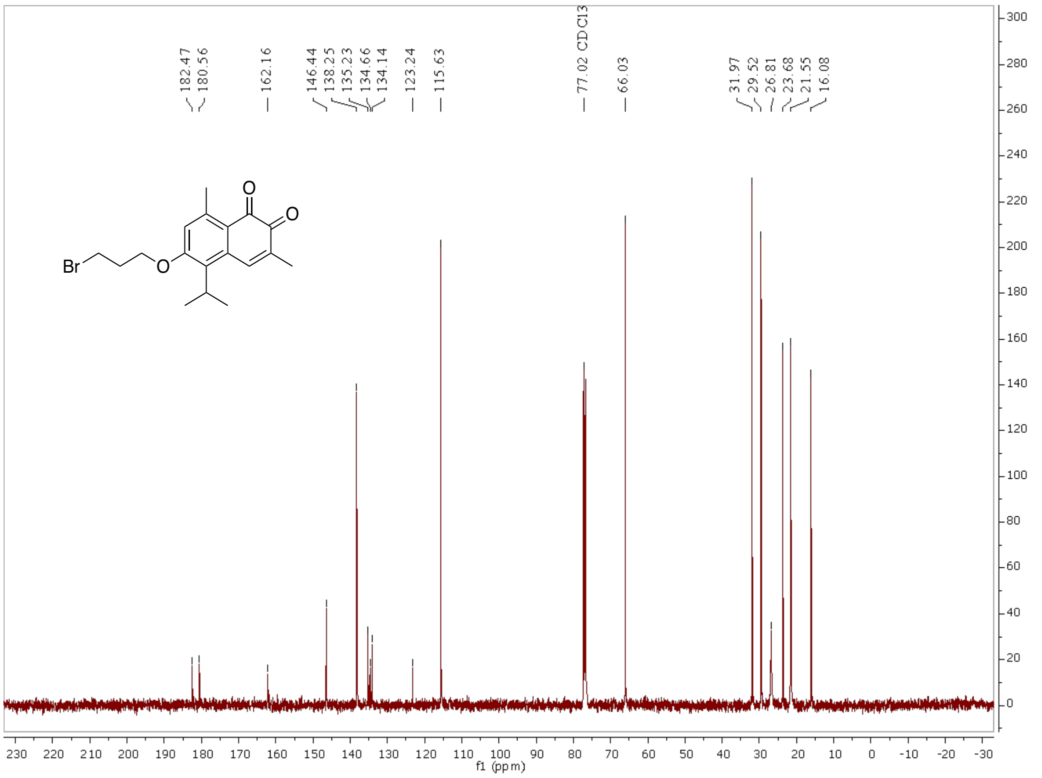

^{13}C NMR of 6-(3-bromopropoxy)-5-isopropyl-3,8-dimethylnaphthalene-1,2-dione (**8**)

F. Compound 9.

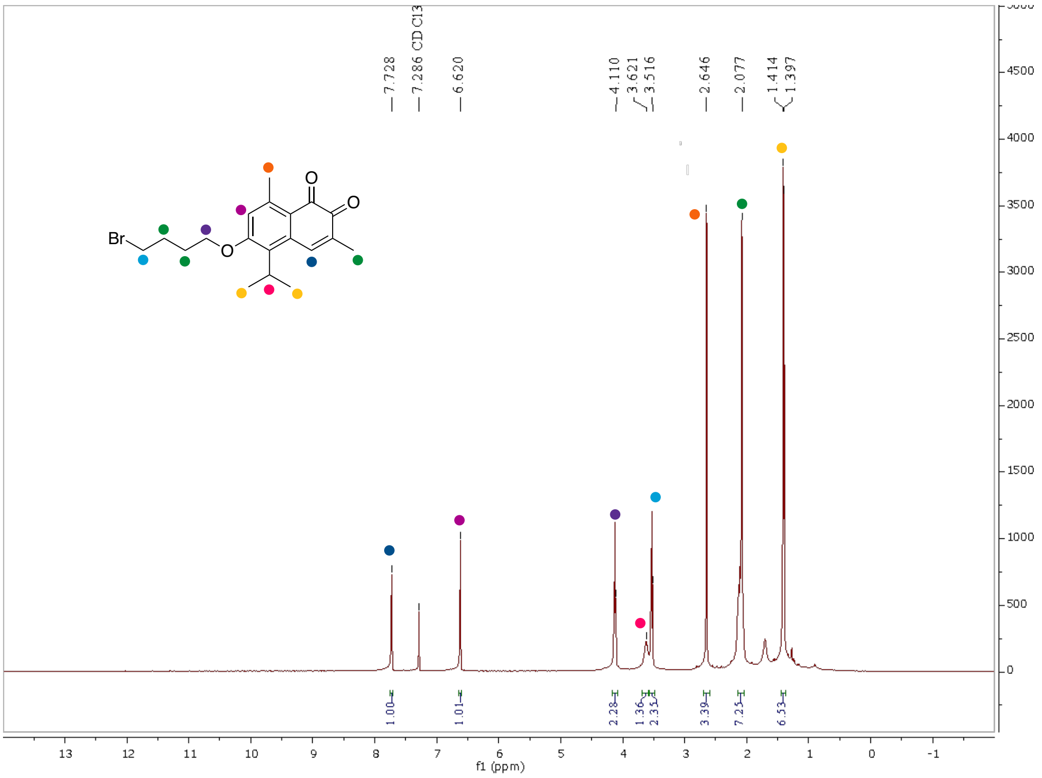

^{1}H NMR of 6-(4-bromobutoxy)-5-isopropyl-3,8-dimethylnaphthalene-1,2-dione (**9**)

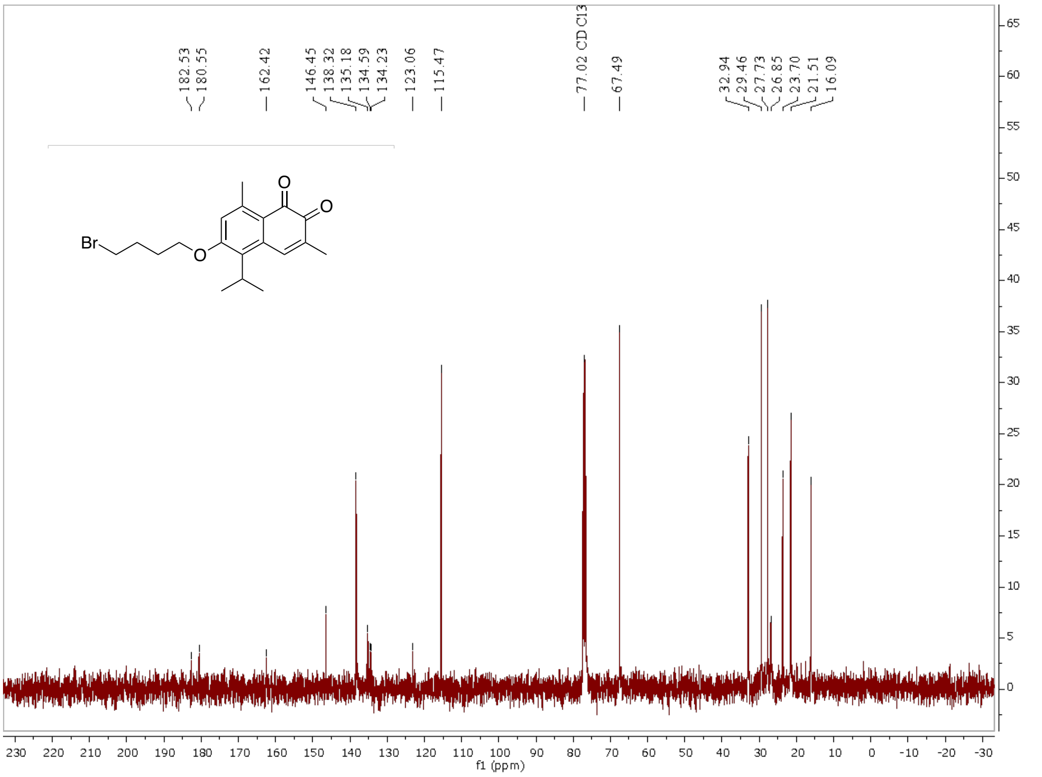

^{13}C NMR of 6-(4-bromobutoxy)-5-isopropyl-3,8-dimethylnaphthalene-1,2-dione (**9**)

G. Compound 10.

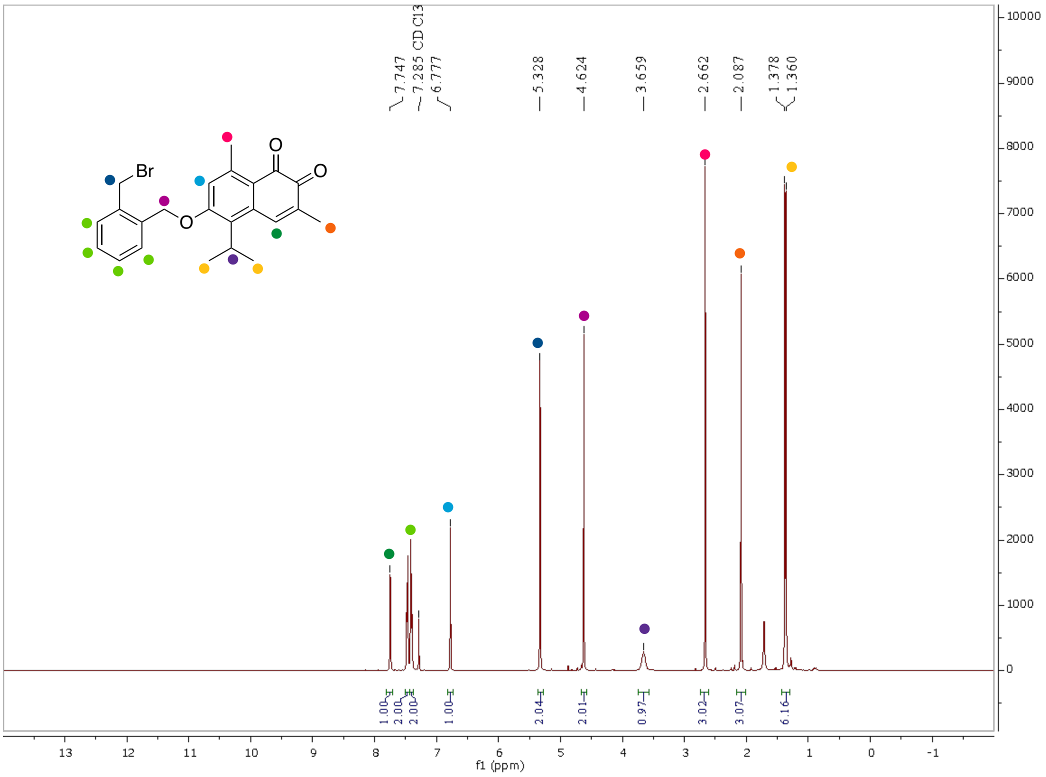

^{1}H NMR of 6-((2-(bromomethyl)benzyl)oxy)-5-isopropyl-3,8-dimethylnaphthalene-1,2-dione (**10**)

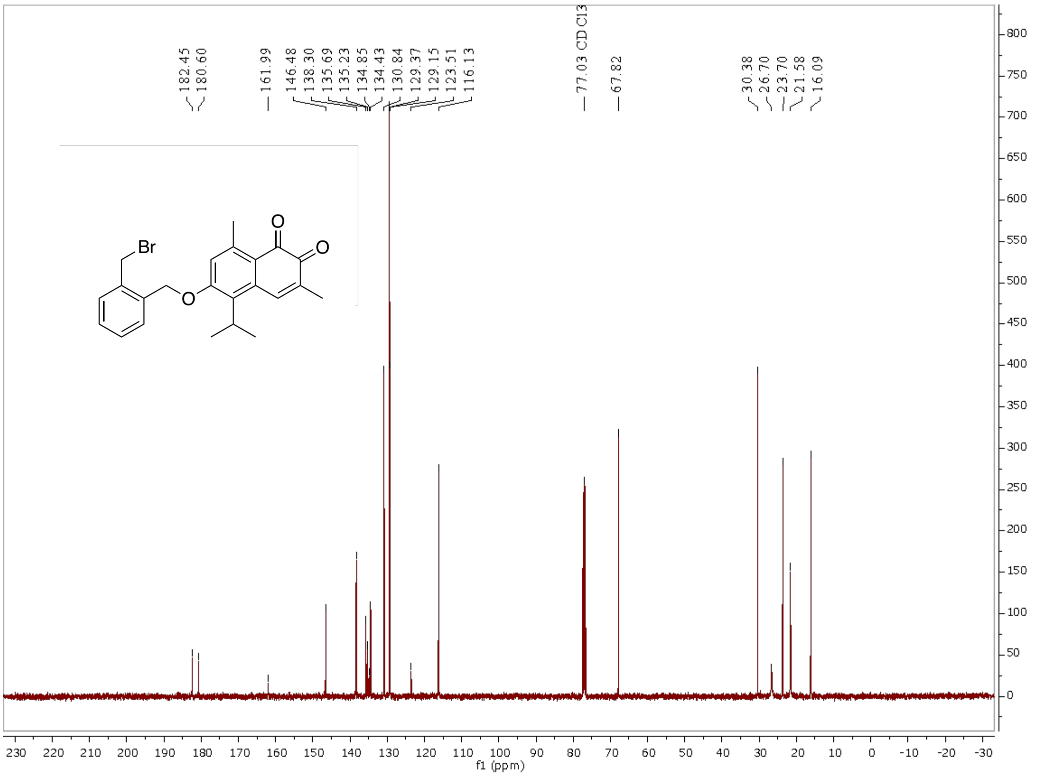

^{13}C NMR 6-((2-(bromomethyl)benzyl)oxy)-5-isopropyl-3,8-dimethylnaphthalene-1,2-dione (**10**)

H. Compound 11.

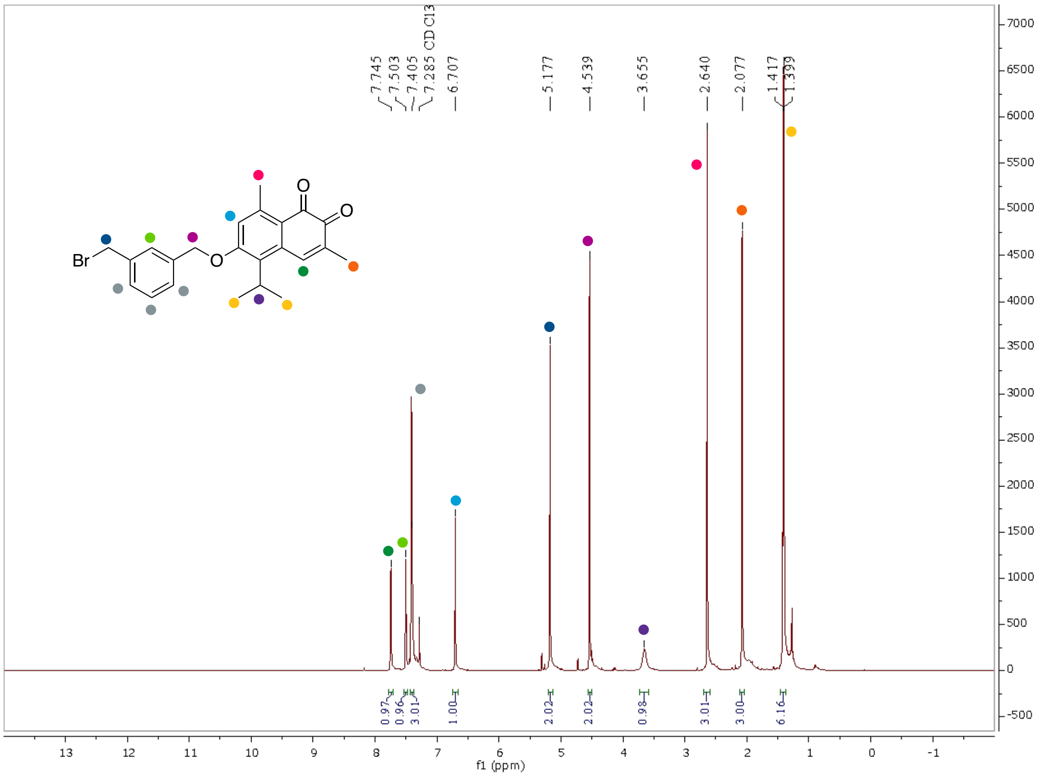

^{1}H NMR of 6-((3-(bromomethyl)benzyl)oxy)-5-isopropyl-3,8-dimethylnaphthalene-1,2-dione (**11**)

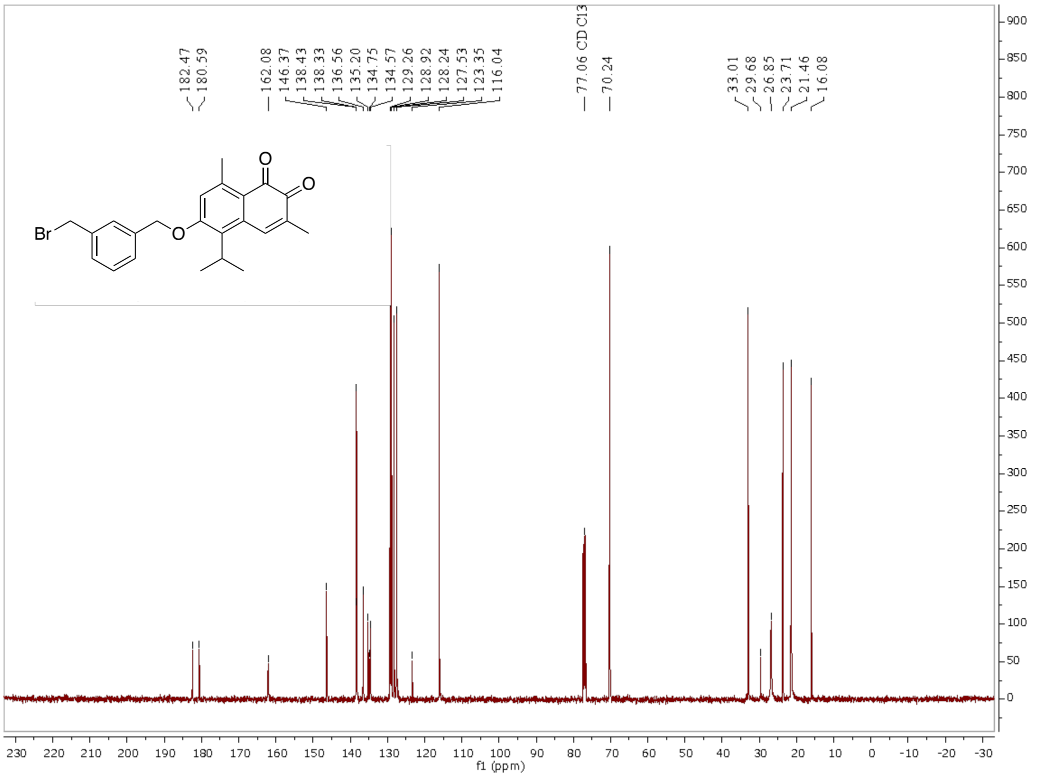

^{13}C NMR of 6-((3-(bromomethyl)benzyl)oxy)-5-isopropyl-3,8-dimethylnaphthalene-1,2-dione (**11**)

I. Compound 12.

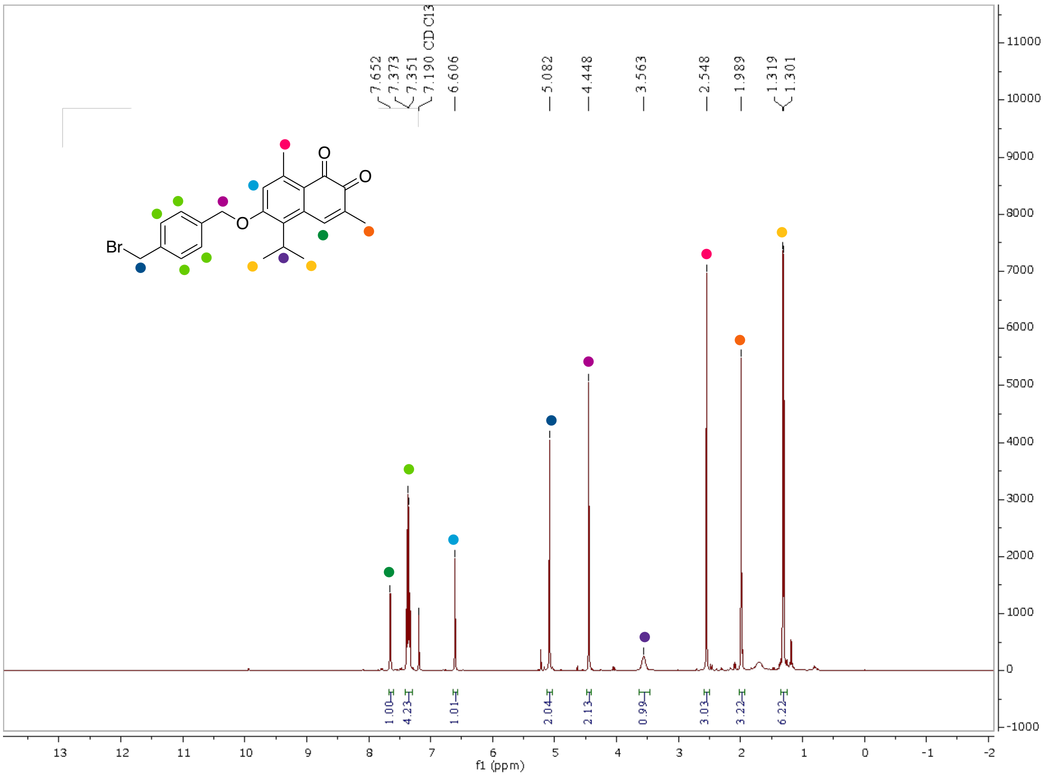

^{1}H NMR of 6-((4-(bromomethyl)benzyl)oxy)-5-isopropyl-3,8-dimethylnaphthalene-1,2-dione (**12**)

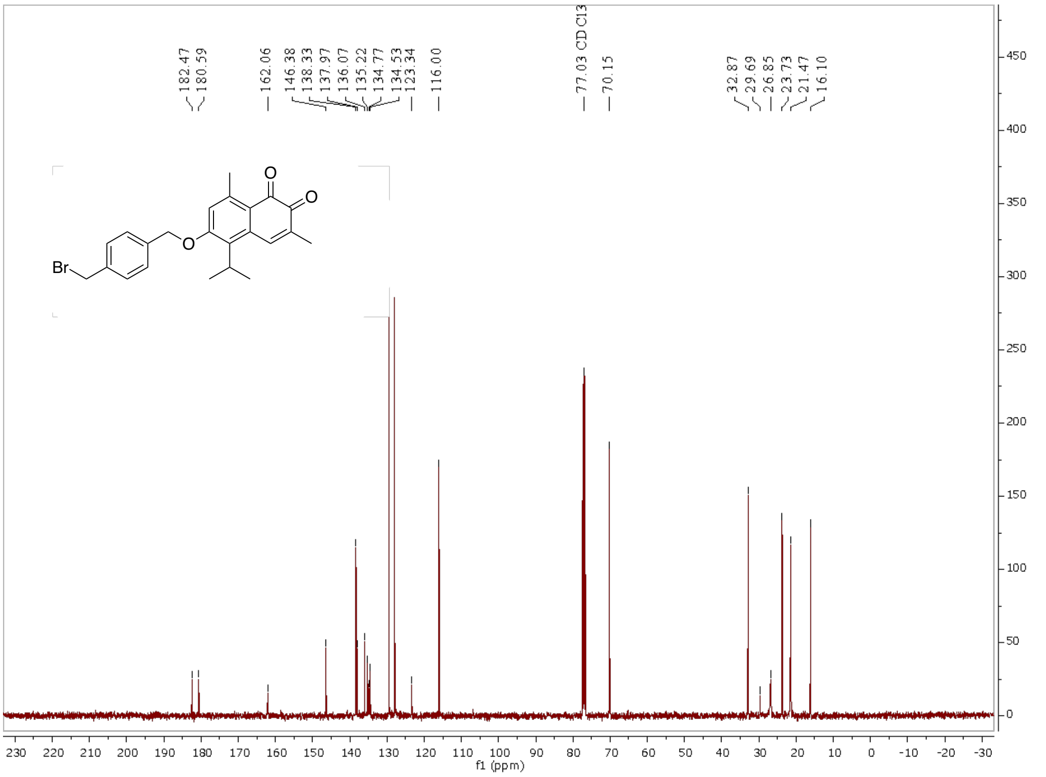

^{13}C NMR of 6-((4-(bromomethyl)benzyl)oxy)-5-isopropyl-3,8-dimethylnaphthalene-1,2-dione (**12**)

J. Compound 13.

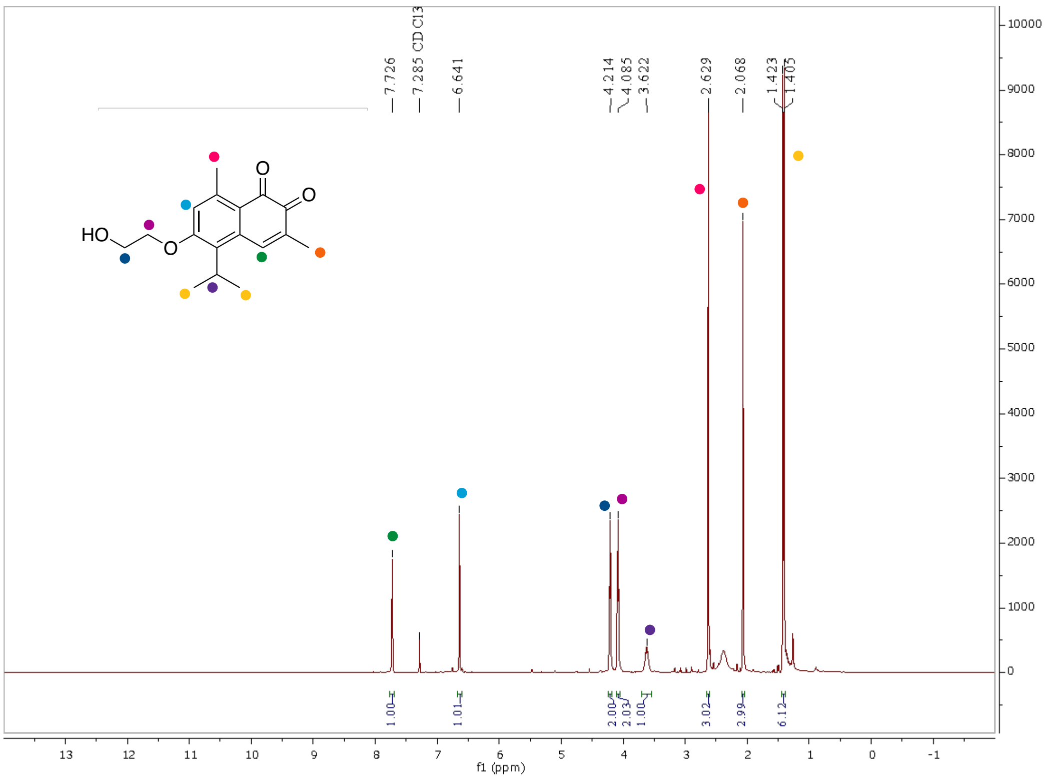

^{1}H NMR of 6-(2-hydroxyethoxy)-5-isopropyl-3,8-dimethylnaphthalene-1,2-dione (**13**)

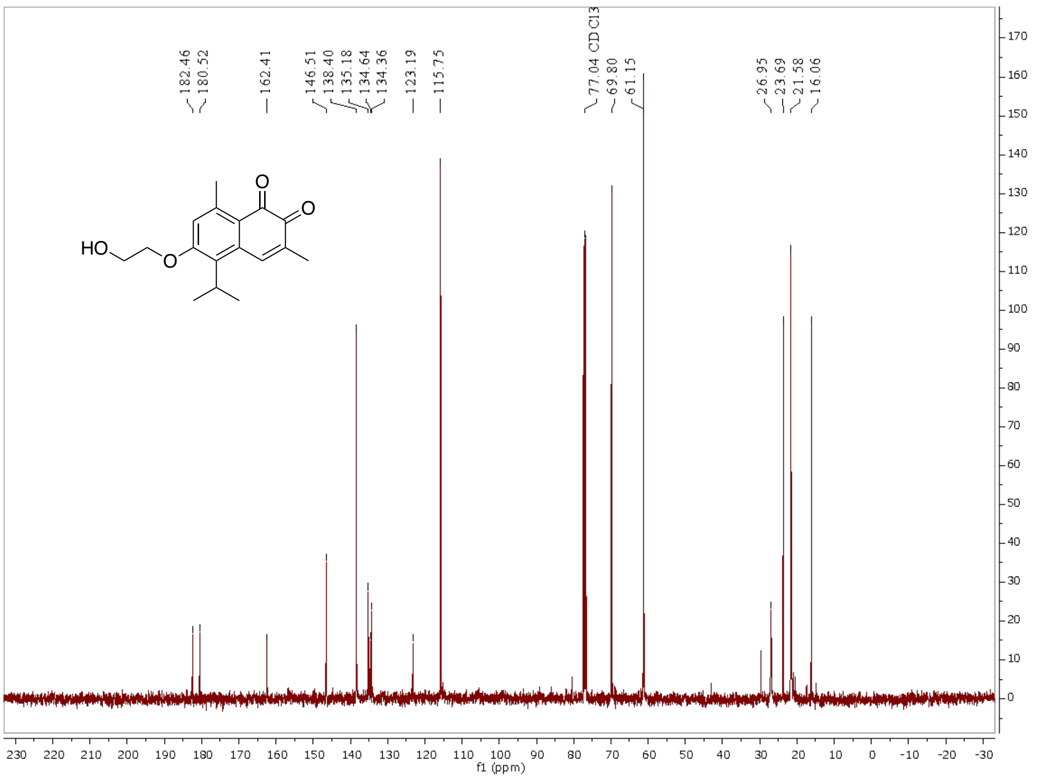

^{13}C NMR of 6-(2-hydroxyethoxy)-5-isopropyl-3,8-dimethylnaphthalene-1,2-dione (**13**)

K. Compound 14.

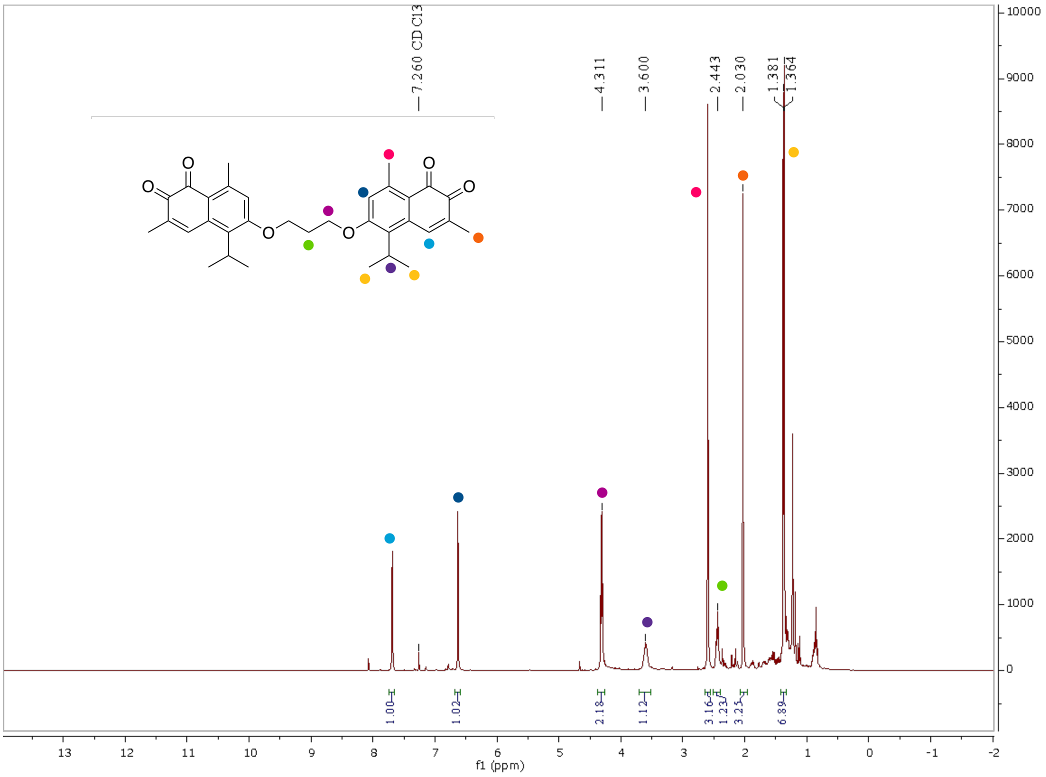

^{1}H NMR of 6,6'-(propane-1,3-diylbis(oxy))bis(5-isopropyl-3,8-dimethylnaphthalene-1,2-dione) (**14**)

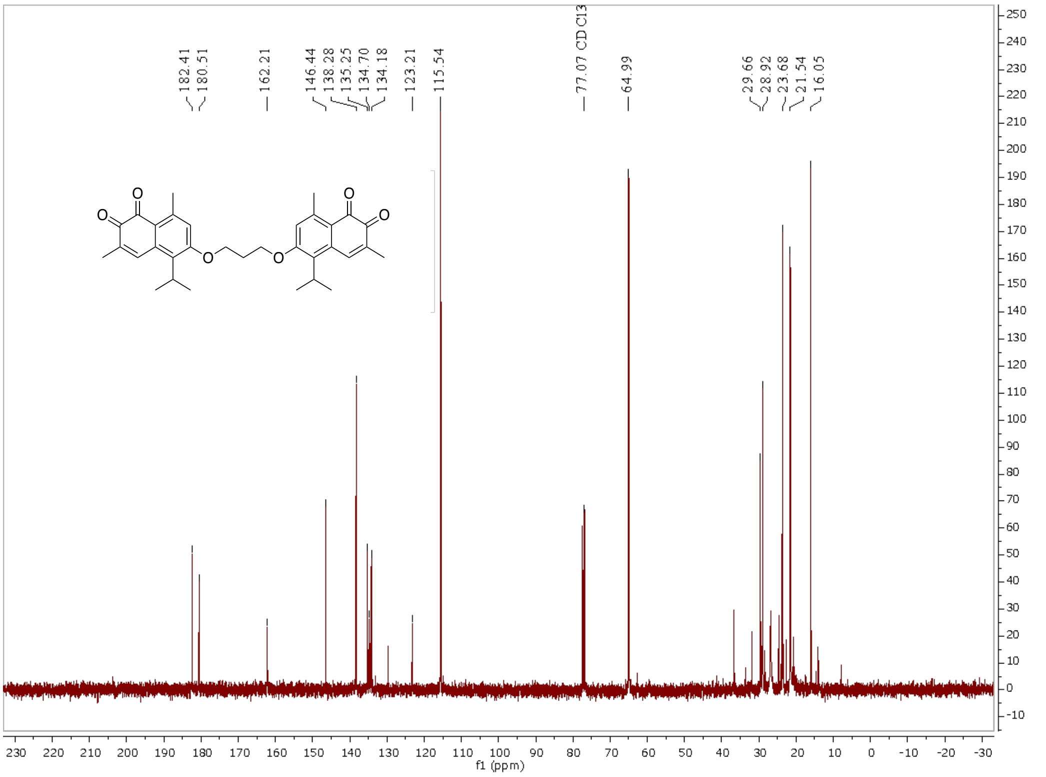

^{13}C NMR of 6,6'-(propane-1,3-diylbis(oxy))bis(5-isopropyl-3,8-dimethylnaphthalene-1,2-dione) (**14**)

L. Compound 15.

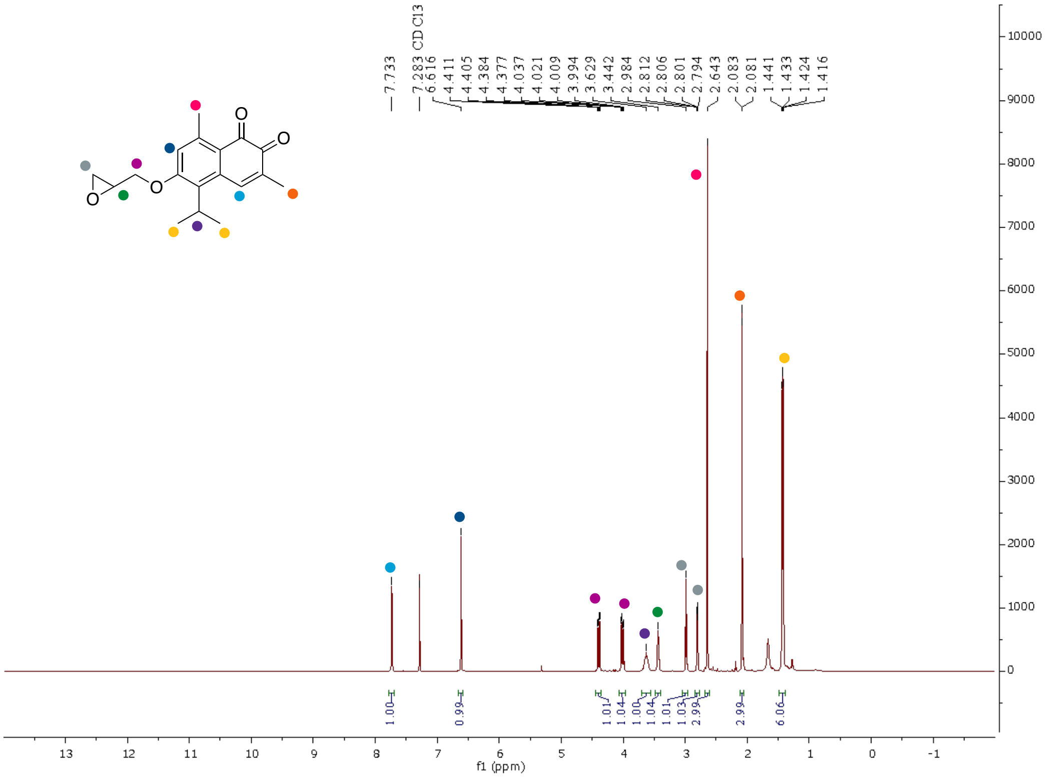

^{1}H NMR of 5-isopropyl-3,8-dimethyl-6-(oxiran-2-ylmethoxy)naphthalene-1,2-dione (**15**)

M. Compound 16.

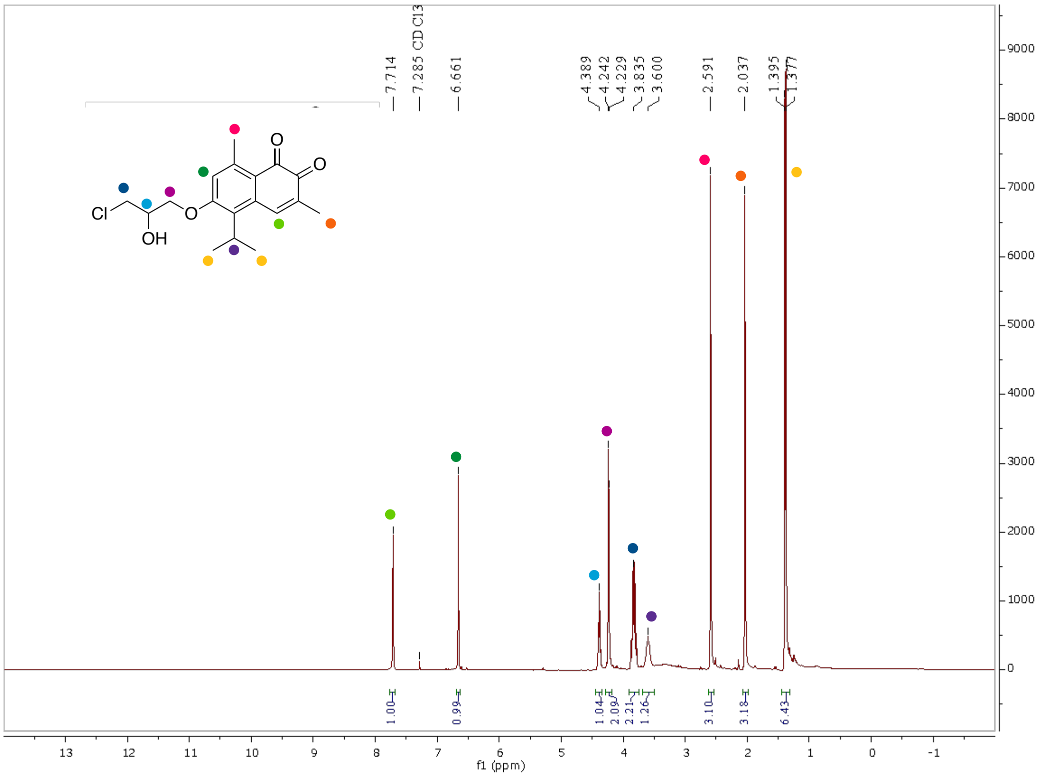

^{1}H NMR of 6-(3-chloro-2-hydroxypropoxy)-5-isopropyl-3,8-dimethylnaphthalene-1,2-dione (**16**)

N. Compound 17.

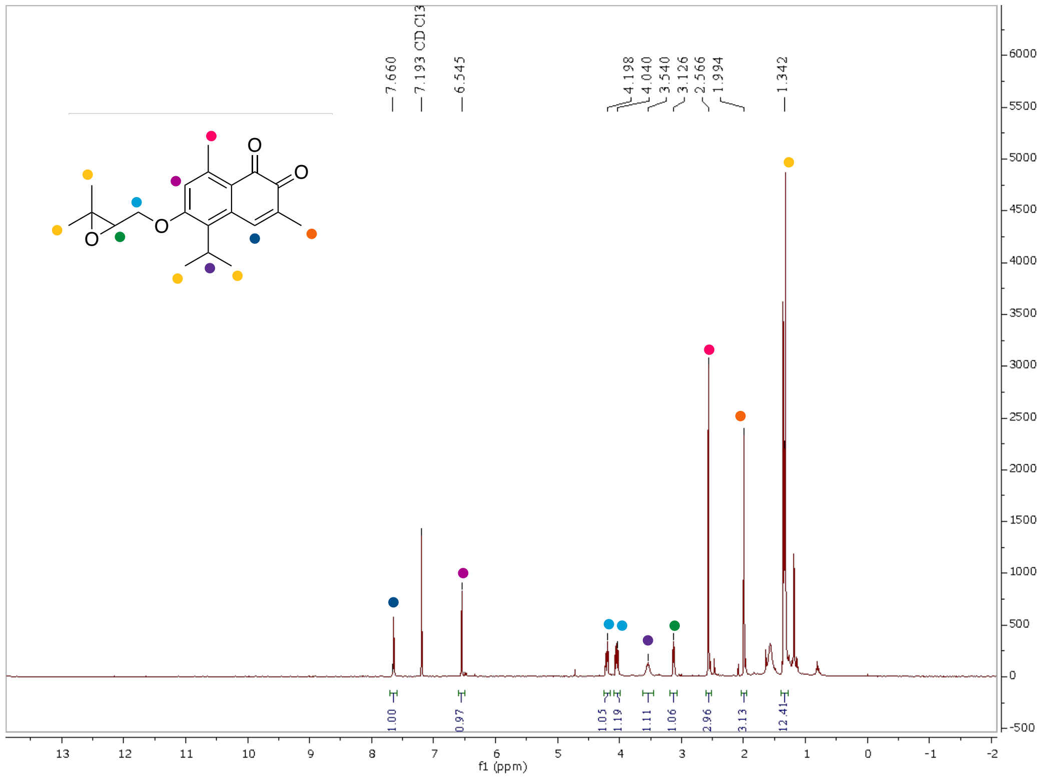

^{1}H NMR of 6-(3,3-dimethyloxiran-2-yl)methoxy)-5-isopropyl-3,8-dimethylnaphthalene-1,2-dione **(17)**

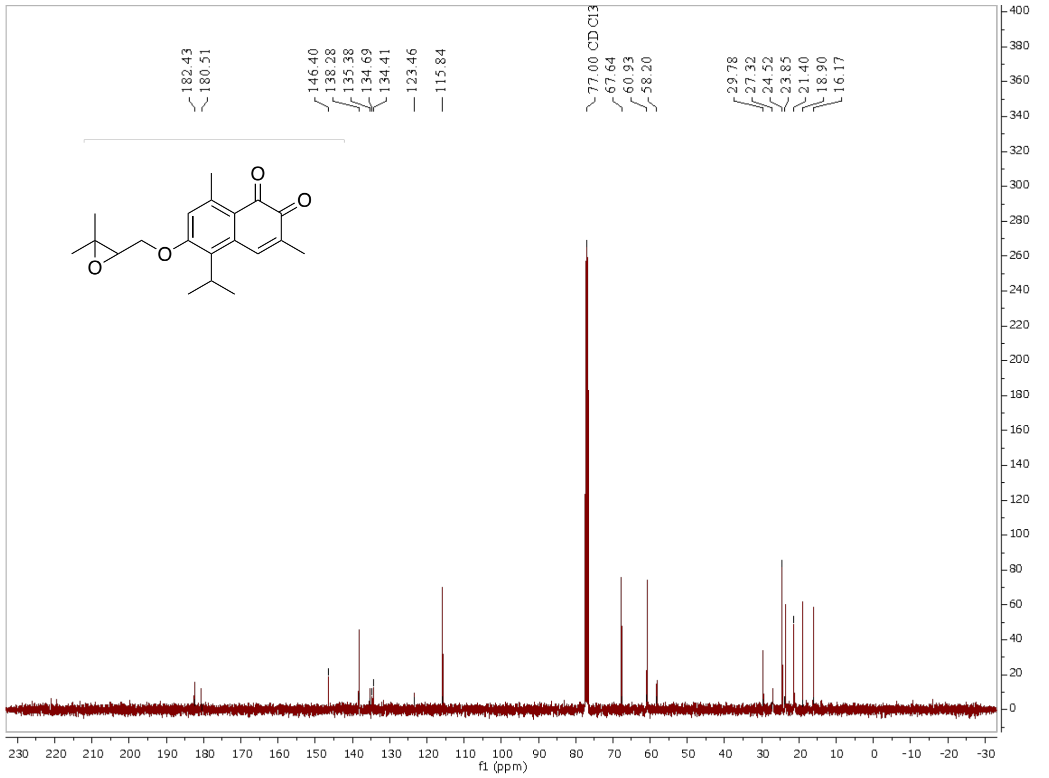

^{13}C NMR of 6-(3,3-dimethyloxiran-2-yl)methoxy)-5-isopropyl-3,8-dimethylnaphthalene-1,2-dione **(17)**

O. Compound 18.

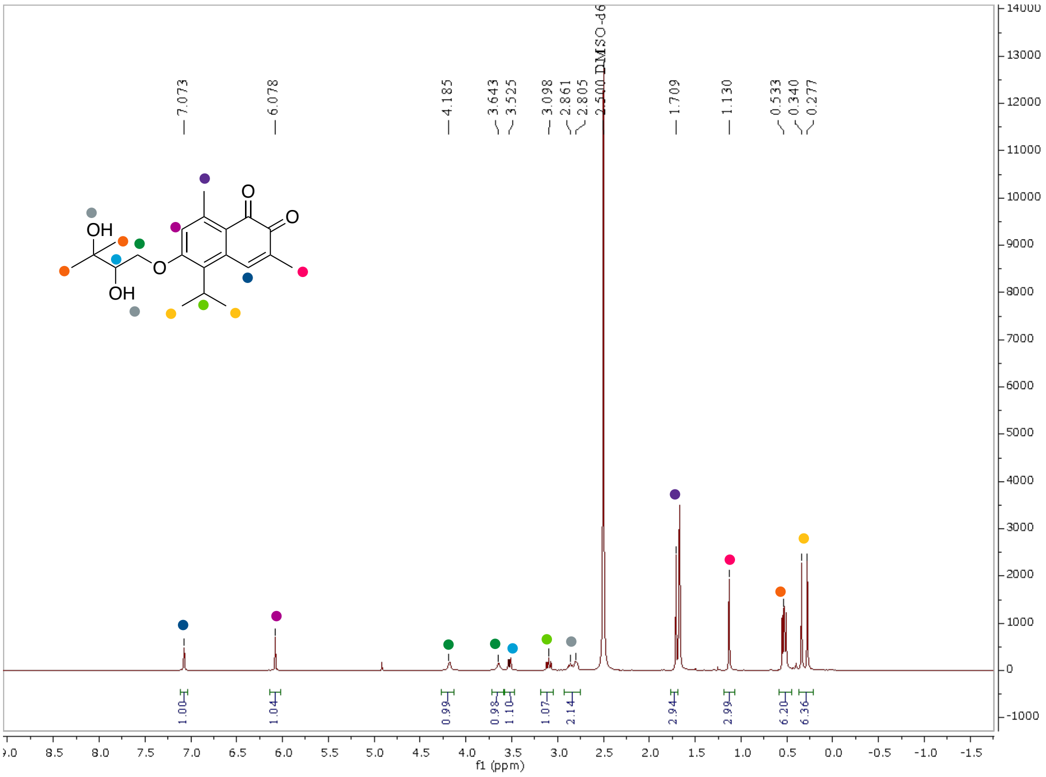

^{1}H NMR of 6-(2,3-dihydroxy-3-methylbutoxy)-5-isopropyl-3,8-dimethylnaphthalene-1,2-dione (**18**)

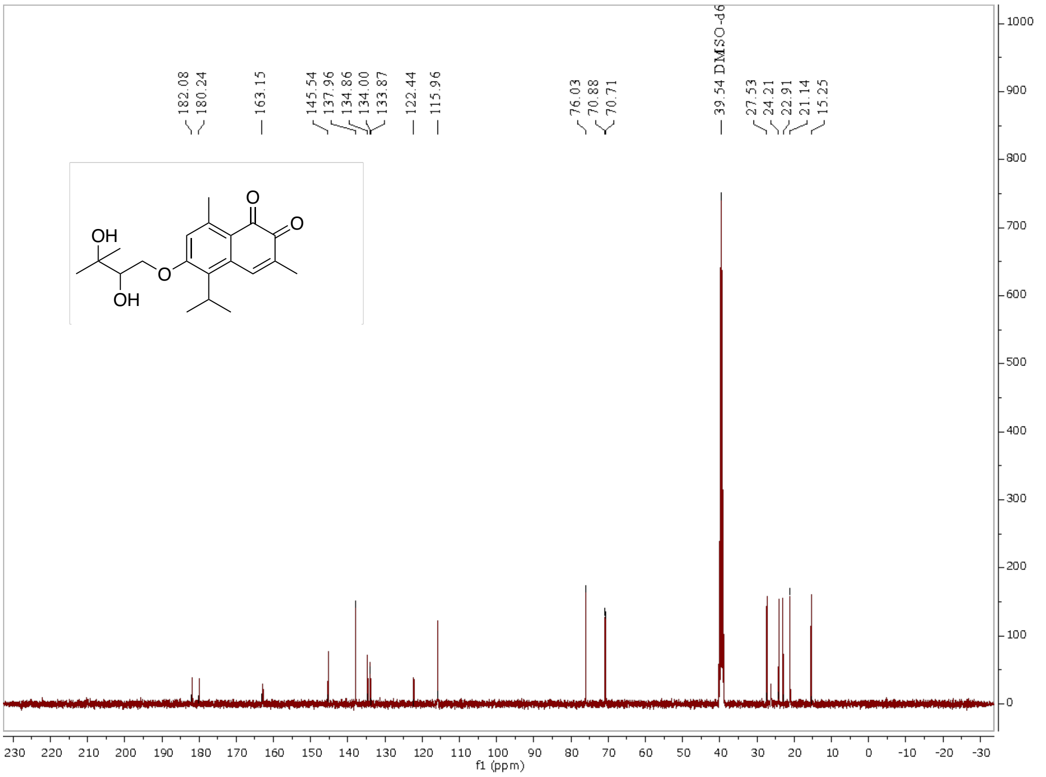

^{13}C NMR of 6-(2,3-dihydroxy-3-methylbutoxy)-5-isopropyl-3,8-dimethylnaphthalene-1,2-dione (**18**)
